# Acute Respiratory Distress Syndrome: Focus on Viral Origin and Role of Pulmonary Lymphatics

**DOI:** 10.3390/biomedicines9111732

**Published:** 2021-11-20

**Authors:** Eleonore Fröhlich

**Affiliations:** 1 Center for Medical Research, Medical University of Graz, 8010 Graz, Austria; eleonore.froehlich@medunigraz.at; Tel.: +43-31638573011; 2 Research Center Pharmaceutical Engineering GmbH, 8010 Graz, Austria

**Keywords:** acute respiratory distress syndrome, pulmonary lymphatics, influenza virus, corona virus, pulmonary edema, acute respiratory distress syndrome (ARDS) treatment

## Abstract

Acute respiratory distress syndrome (ARDS) is a serious affection of the lung caused by a variety of pathologies. Great interest is currently focused on ARDS induced by viruses (pandemic influenza and corona viruses). The review describes pulmonary changes in ARDS and specific effects of the pandemic viruses in ARDS, and summarizes treatment options. Because the known pathogenic mechanisms cannot explain all aspects of the syndrome, the contribution of pulmonary lymphatics to the pathology is discussed. Organization and function of lymphatics in a healthy lung and in resorption of pulmonary edema are described. A future clinical trial may provide more insight into the role of hyaluronan in ARDS but the development of promising pharmacological treatments is unlikely because drugs play no important role in lymphedema therapy.

## 1. Introduction

Adult acute respiratory distress syndrome (ARDS) was first described as a non-cardiogenic pulmonary edema and is currently defined according to the “Berlin Definition of ARDS” as the acute onset of hypoxia and bilateral pulmonary opacities not fully explained by a cardiac cause [1]. Acute onset is specified to be within 1 week of a precipitating illness and hypoxia is determined by a partial pressure of oxygen (PaO_2_) to fraction of inspired oxygen (FiO_2_) ratio less than or equal to 300 mm Hg while receiving a minimum of 5 cm H_2_O of positive end-expiratory pressure (PEEP). Although lung tissue of infants is less prone to inflammation and fibrosis and the relative amount of endogenous surfactant is higher in children than in adults, inflammation, cellular damage, and surfactant dysfunction occur in a similar way as in adults [2]. Despite the similarities to adult ARDS, the Pediatric Acute Lung Injury Consensus Conference (PALICC) published, in 2015, a pediatric-specific definition for ARDS [3].

Adult ARDS can develop in various pathological conditions and is classified as “direct” or “indirect” based on the underlying pathology [4]. Affections of the lung (pneumonia, aspiration, and pulmonary contusion) cause direct ARDS, extrapulmonary (systemic) diseases (non-pulmonary sepsis, non-thoracic trauma, and transfusion) indirect ARDS. The majority of ARDS cases are caused by severe pneumonia (30–50%), sepsis (25–30%), and severe trauma 10–25%. Bacteria-induced ARDS (*Streptococcus pneumonia, Staphylococcus aureus*) was more frequent than viral-induced ARDS (influenza A) or fungal ARDS (*Pneumocystis jirovecii*). Diffuse alveolar damage (DAD) was seen in 45% of patients, while 55% of the lungs demonstrated various other histopathologic findings. In direct ARDS, usually more DAD, alveolar collapse, fibrin deposition, and alveolar wall edema is seen than in indirect ARDS. The largest cross-sectional study including 50 countries (LUNGSAFE) reported that ARDS incidence in intensive care unit (ICU) patients was higher in Europe (0.48 cases/ICU bed over 4 weeks) und the United States (0.46 cases) than in Africa (0.32 cases), South America (0.31 cases), and Asia (0.27) [5]. The incidence of ARDS is lower in 15–19-year-old individuals (16/100,000 person-years) compared to 75–84-year-old persons (306/100,000 person-years) [6]. Among the hospitalized patients, there was, however, no clear difference regarding admission to ICU, ventilation length, and length of hospitalization between patients younger or older than 65 y [7]. Neonatal ARDS is due to immaturity of the lung, and standard treatment consists of the administration of an exogenous surfactant. Clinical trials reported variable efficacy, which appears to be due to the fact that an exogenous surfactant is quickly inactivated by phospholipases and that commercially available surfactants lack anti-inflammatory compounds of natural surfactants, such as surfactant proteins A and D or dioleoyl-phosphatidylglycerol. In contrast to neonate ARDS, there is no standardized pharmacological treatment for adult ARDS.

Research on adult ARDS intensified recently because in December 2019 pulmonary infections caused by a new virus, the severe acute respiratory syndrome corona virus 2 (SARS-CoV-2), occurred. SARS-CoV-2 was classified as a pandemic in March 2020 and, by the end of October 2021, caused almost 5 million deaths worldwide. ARDS developed in 42% of patients with SARS-CoV-2-induced pneumonia [8]. Due to the pandemic, ARDS cases in the United States rose from 495,655 in 2017, to 550,371 in 2020 [9]. There is a great need to better understand the syndrome and to identify critical parameters and potential prognostic markers for survival. Several studies investigated mortality rates from ARDS. They varied from 15 to 72% with an overall pooled mortality of 43% and showed a trend for decrease between 1994 to 2006 [10]. The authors concluded that the main factor was the improved lung-protective ventilation but that also better timing and rationalization of therapeutic interventions, glucose control, hygienic measures, and better sepsis management played a role. By contrast, pharmacological treatment was less effective (see [11]). Also, the large LUNGSAFE study, published in 2016, reported mortality of 40%, highlighting the need for better treatments [5].

This review will describe pulmonary changes in ARDS, the two common types of viruses that induce ARDS including their differences in morphology and replication cycle, and list currently available treatments. The role of pulmonary lymphatic vessels will be discussed as an additional parameter in ARDS by describing the architecture and function of the pulmonary lymphatic system.

## 2. Lung Changes in ARDS

**Figure 1:**
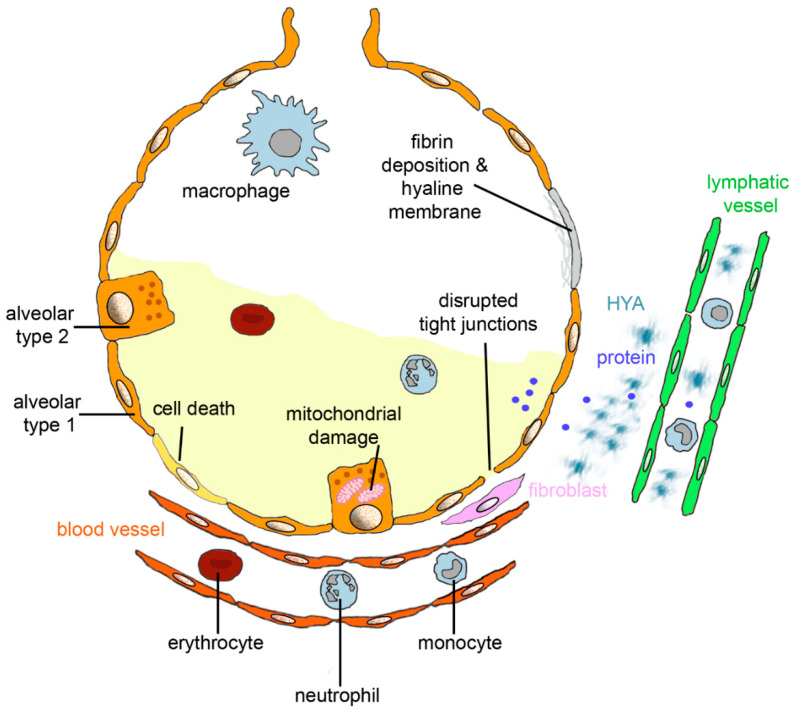
Alveolar changes in acute respiratory distress syndrome (ARDS). Diffuse alveolar damage (DAD) is characterized by death of alveolar epithelial cells; disruption of tight junction between alveolar type 1 cells; and mitochondrial damage and invasion of erythrocytes, monocytes, and neutrophilic granulocytes from blood vessels. Interstitial fluid and protein accumulate in the alveolus, and fluid, protein, and hyaluronan (HYA) in the interstitium. If fluid and proteins are not removed from the alveolar lumen by epithelial sodium channels and Na/K-ATPase or endocytosis by the alveolar epithelium and fluid, then proteins and HYA uptake into lymphatic vessels, and fibrin deposition and formation of hyaline membranes occurs. Excessive formation of collagen by fibroblasts leads to fibrosis.

ARDS irrespective of the underlying disease causes a spectrum of pathological changes mainly in the alveolar parts of the lungs. The alveoli are the most important part of the lung for gas exchange and, therefore, the alveolar barrier has to be very thin. The lining epithelia of the alveolar surface is composed of 95% of the flat alveolar epithelial type I (AT1) cells (Figure 1). The AT2 cells are slightly taller and serve for the production of surfactant to decrease lung surface tension and for replacement of damaged AT1 cells. Together with the liquid layer on top of the epithelium and the endothelium of the blood vessels, the barrier for gas exchange has a maximum thickness of 2.2 µm [12]. Other important cells at the alveolar barrier are the alveolar macrophages, which recycle pulmonary surfactant; remove foreign particles and debris from the lung surface; and keep the lungs in a quiet, not inflamed, state. More detailed description of the lung architecture and morphology is available elsewhere (e.g., [13]).

The course of ARDS can be divided into exudative, proliferative, and fibrotic phase [14]. The increase of pulmonary water content is termed pulmonary edema and is one of the most important features of the exudative phase of ARDS. Lung tissue, cells, blood, and interstitial space consists of 80% of water [15]. If the normal amount of interstitial water (35%) increases to 50%, individual alveoli will fill with fluid. Fluid accumulation not only in the alveoli but also in the interstitial space of the lungs hinder gas exchange [16]. Intrinsic contractility of lymph vessels as well as the inspiration and expiration of lymphatic valves determine lymphatic flow, but fluid transport capacity appears to be low. Furthermore, lymphatic vessels may be compressed when the interstitial pressure increases. Fluid accumulates first in the hilar region and in sheaths around the large pulmonary vessels, where pressure is highest and interstitial compliance lowest. Hydrostatic (cardiogenic) and increased permeability pulmonary edema (ARDS) show similar radiologic findings, such as widened vascular pedicle, pleural effusion, peribronchial cuffs, and septal lines [17]. Although the frequency of these findings differs between the two forms of ARDS, chest radiography is not able to reliably differentiate between them. The exudative phase lasts for about 7 days and is characterized by eosinophilic depositions (hyaline membranes), alveolar hemorrhage, accumulation of white blood cells (mainly neutrophilic granulocytes), fibrin deposits, and alveolar collapse. Intercellular junctions are disrupted in ARDS due to alveolar cell damage, cell loss, and invasion of neutrophils. In the proliferative phase (3 weeks), hyperplasia of AT2 cells and interstitial fibrosis takes place. In the fibrotic phase, fibrosis continues and loss of alveolar structure and emphysema occur. Clinically oriented descriptions of ARDS discriminate between exudative (edema), transition (hyaline membrane formation), proliferative (inflammation and fibroplasia) and fibrotic (progressive, stable fibrosis, or resolution) phase [18]. According to another classification, there are two only phases, exudative and fibroproliferative phase, where the proliferative phase consists of a subacute phase of 7–14 days with reabsorption of pulmonary edema, proliferation of AT2 cells, infiltration with fibroblasts, and collagen deposition. The subsequent chronic phase of undetermined length involves resolution of the neutrophil infiltrate, accumulation of macrophages and mononuclear cells in the alveoli, and additional fibrotic changes [19]. Increased numbers of fibroblasts in the interstitial space issue cause excessive collagen production and may lead to remodeling and lung pulmonary atrial hypertension (PAH). PAH is defined as >20 mm Hg as mean pulmonary arterial pressure. Acute increase in pulmonary artery pressure due to vasoconstriction is commonly seen in ARDS and most likely contributes to ventilation-perfusion mismatch, which is a cause of hypoxemia. The prevalence of acute PAH in ARDS has been indicated as 46.6–92% [20]. PAH in ARDS did not increase mortality of ARDS, which was 36.6% for both groups [21]. PAH after recovery from ARDS was also reported in 60% of survivors with SARS-CoV-2 [22]. Increased circulating endothelin 1 (ET-1) levels and local deposition of hyaluronan/hyaluronic acid (HYA) in the lung parenchyma are indicators for PAH [23]. Relevant for the development of PAH are accumulation and activation of neutrophils in the lung microvasculature with release of proteases, reactive oxygen species, pro-inflammatory cytokines, and pro-coagulant molecules [19]. It is generally accepted that the first 7 days after the onset of ARDS are the critical period, where the main treatment goal is to reduce DAD as well as possible, because it is associated with higher mortality. Vascular remodeling and proliferation of smooth muscle cells through the release of ET-1 occlude the pulmonary vasculature, and fibrocellular obliteration of the microvasculature may subsequently leads to manifesting PAH. Pulmonary fibrosis and PAH are linked because PAH is a quite common complication of pulmonary fibrosis with a reported occurrence from 32% to 85% [24]. Lung fibrosis occurred after less than 1 week in 4% of the patients [25], but typically (61% of patients) fibrosis develops after a duration of greater than 3 weeks.

ARDS is not an isolated pathology of the lung but is linked to general hypoxemia, dysregulated hemostasis, and multiorgan dysfunction syndrome (MODS), which affects renal, hepatic, gastrointestinal, central nervous, and cardiovascular system [26]. It is hypothesized that ARDS is not the cause of MODS but ARDS and MODS are correlated syndromes promoted by vascular microthrombotic disease (VMTD) [27].

Instead of increasing fibrosis and tissue remodeling, resolution may occur. Apical epithelial sodium channels (ENaC) and basolateral Na/K-ATPase of AT1 and AT2 cells can transport fluid from the lumen of the alveoli to the interstitial space. Proteins are absorbed by endocytosis of alveolar epithelial cells. Lymphatics and microcirculation in the healthy lungs subsequently remove the fluid together with macromolecules such as proteins and HYA, from the interstitial space. In ARDS, resorption is delayed because decreased ENaC and cystic fibrosis transmembrane conductance regulator (CFTR) activity of cells infected with swine flu (influenza A virus H1N1) [28] and downregulation of Na/K-ATPase by hypoxia hinder fluid resorption. Proliferation of cytokeratin 5 positive (KRT5+) basal cells to replace alveolar cell loss is decreased by hypercapnia, and surfactant dysfunction induced by proteinaceous fluid in the alveoli results in atelectasis [23]. Virus-induced ARDS has gained less attention in the past because it is not common in nonimmunocompromised patients [29]. Corona viruses and pandemic influenza A viruses, however, have a significantly higher frequency of ARDS [30]. Characteristics of SARS-CoV-2-induced ARDS in contrast to ARDS caused by other pathologies were listed as follows: patients display little breathlessness despite marked hypoxemia; lung compliance is well preserved; hypoxemia is associated with large intrapulmonary shunt; and the benefit of prone ventilation is larger than for typical ARDS [31].

### 2.1. Virus-Induced ARDS

**Figure 2:**
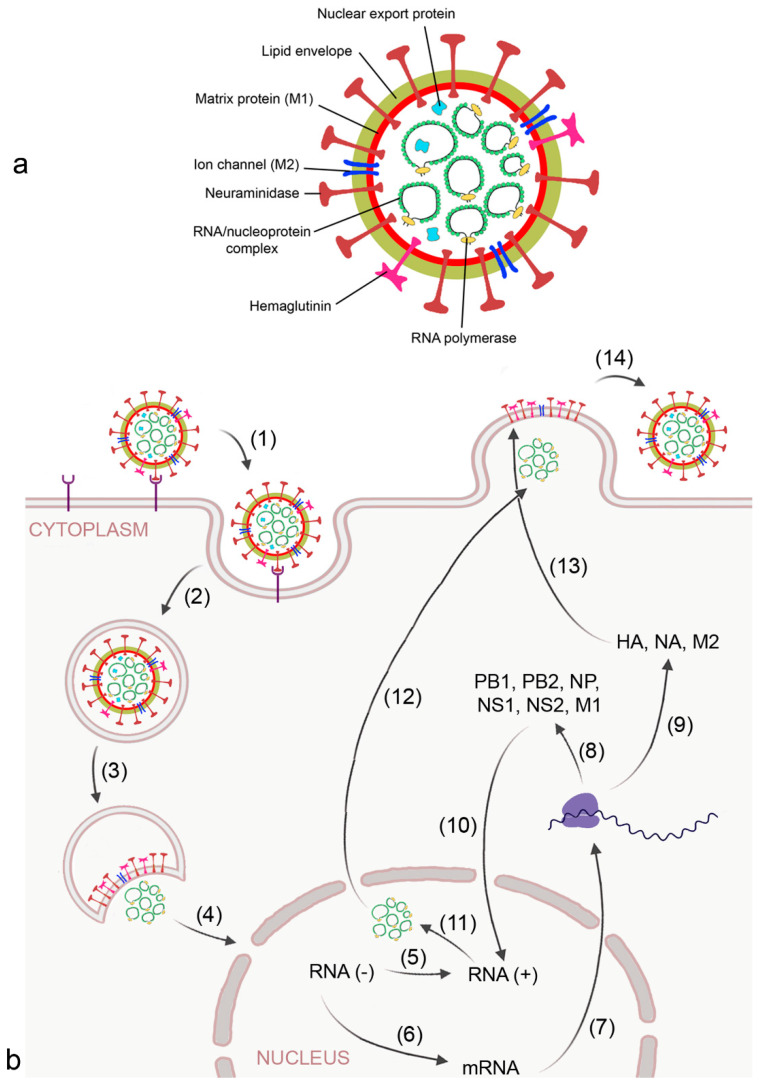
Morphology (**a**) and replication cycle (**b**) of influenza A virus. Replication includes the following steps: (1) binding of the virus to sialic acid-containing surface receptors by viral hemagglutinin (HA); (2) endocytotic uptake; (3) release of the RNA/ nucleoprotein complex; (4) transport of the ribonucleoproteins into the nucleus; (5) replication of the RNA strand by viral RNA polymerase into the complimentary + strand; (6) transcription of the RNA strand into mRNA by viral RNA polymerase; (7) export of mRNA from the nucleus; (8) synthesis of viral RNA polymerase subunits polymerase basic (PB) 1 and 2, matrix protein 1 (MP1), nucleoprotein (NP), and non-structural (NS)1 and 2 for reconstruction of the RNA/nucleoprotein complex; (9) synthesis of HA, neuraminidase (NA), and matrix protein 2 (M2) at the host ribosome and endoplasmic reticulum;(10) transport of PB1, PB2, M1, NP, NS1, NS2 into the nucleus; (11) formation of the nucleoprotein complex; (12) transport of the nucleoprotein complex out of the nucleus by viral nuclear export protein; (13) integration of HA, NA, and M2 into the budding plasma membrane, and (14) release of the virion by NA action.

Pandemic influenza A viruses and corona viruses are the most relevant viruses that may cause ARDS. Influenza A is a negative sense RNA virus that expresses 11 proteins. The RNA of the virus is surrounded by lipid envelope, matrix protein 1 with integrated ion channel matrix protein 2, and neuraminidase (NA) and hemagglutinin (HA) at the surface (Figure 2a). HA and NA are the most relevant proteins for infection and propagation of the virus [32]. The viral RNA is coated with nucleoprotein and attached to RNA-dependent RNA polymerase. Further, the virus possesses nuclear export proteins. The low fidelity proofreading capacities of RNA polymerase is the reason for mutations [33], with cumulative changes in HA and NA leading to antigen drift (alteration of fitness for human infection) and re-assorting of HA from avian reservoirs to antigen shifts resulting in “new” viruses to which the population has no specific immunity. Avian species are the reservoir for influenza A viruses, including 16 HA and 9 NA types known in humans, and 2 additional HA and 1 NA in bats.

The virus binds to sialic acid-containing surface proteins for uptake by endocytosis. After the virion is endocytosed by the alveolar epithelial cell and reaches the lysosome, activation of the proton selective matrix protein-2 (M2) viral channel leads to proton entry and dissociation of the ribonucleoprotein complex [32] (Figure 2b). In the nucleus of the host cell, mRNA and viral RNA is synthesized according to the viral RNA template. Progeny viral ribonucleoprotein (RNP) cores are generated in the cytosol and, together with the viral surface proteins HA and NA and other proteins, are concentrated in lipid rafts at the plasma membrane. After budding, the virion is bound to the plasma membrane by interaction of HA with sialic acid residues and released from the host cell upon action of NA.

**Figure 3:**
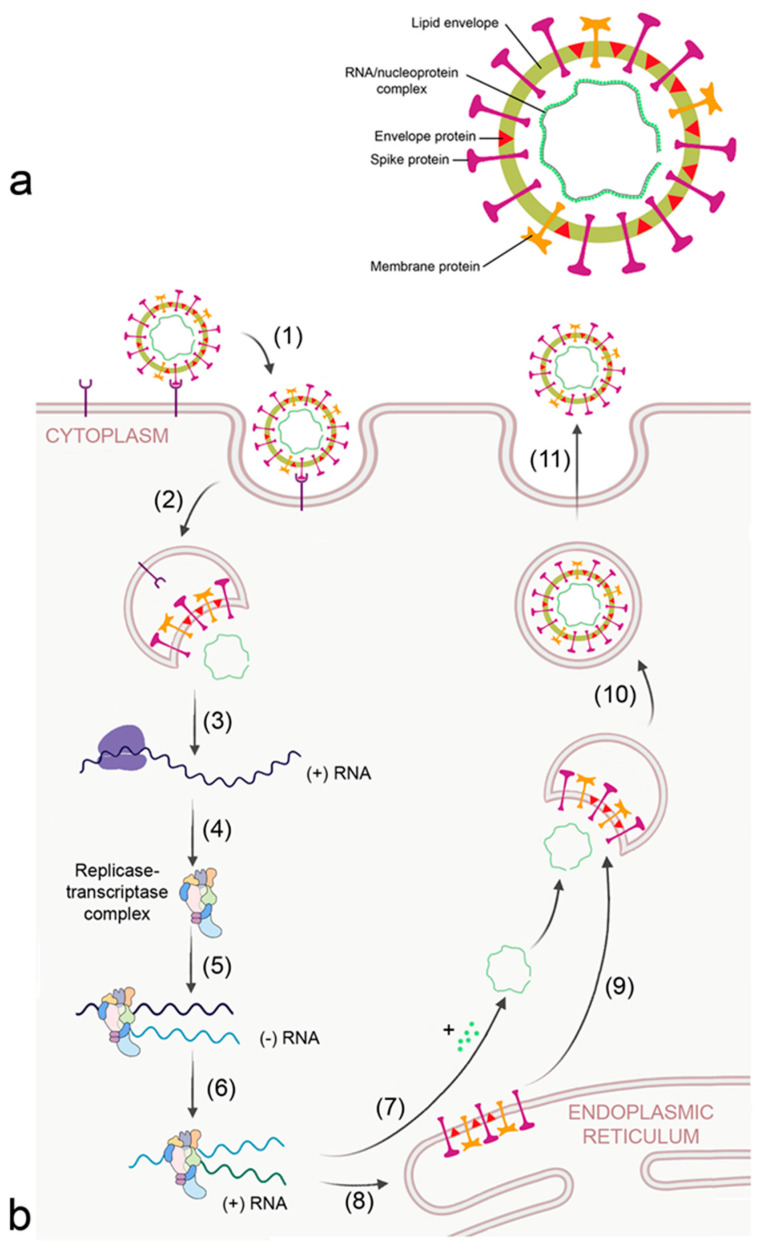
Morphology (**a**) and replication cycle (**b**) of corona virus. The replication cycle consists of the following steps: (1) binding to cellular receptors; (2) cleavage of the virion from the receptor either by transmembrane protease serine subtype 2 (TMPRSS2) at the membrane or by cathepsin L in the endosome; (3) release of RNA and translation of viral RNA to ribosomal proteins by the host ribosomes; (4) formation of the viral replicase-transcriptase complex; (5) generation of the - RNA strand; (6) transcription of + RNA strand from the RNA template; (7) formation of the nucleocapsid; (8) translation of viral proteins; (9) insertion of envelope, spike, and membrane proteins into the ER–Golgi intermediate compartment (ERGIC) for virion assembly; (10) encapsulation of the virion into Golgi vesicles; and (11) secretion of the virion.

The other viruses causing ARDS are pandemic corona viruses. They circulate in non-human species, mainly bats and rodents, and are classified as alpha- and betacorona viruses. Seven viruses have been identified, four causing mild seasonal infections and three (MERS-CoV, SARS-CoV and SARS-CoV-2) severe illness [33]. Coronaviruses are positive-sense single-stranded RNA viruses and have the largest genome of any RNA virus. Ribosomal frame shifting and generation of subgenomic RNA for easy recombination confer the ability to develop novel host specificity. The positive sense RNA can serve as mRNA and is directly translated by the host into viral proteins. The general composition of corona viruses is shown in Figure 3a. Viral RNA is surrounded by a membrane containing envelope, membrane, and spike proteins. The RNA/nucleoprotein complex is arranged as one large ring.

Corona viruses bind to cellular receptors typical for each corona virus and are endocytosed by the host (Figure 3b). Angiotensin-converting enzyme 2 (ACE2) and dendritic cell-specific ICAM-3 grabbing non-integrin (DC-SIGN/CD209) are the receptors for SARS-CoV [34]. MERS-CoV binds to dipeptidyl peptidase 4 (DPP4/CD26). Receptors that were reported to facilitate cellular uptake of SARS-CoV-2 include ACE2, C-lectin type receptors, toll-like receptors (TLR), neuropilin 1 (NLP1), and non-immune receptor glucose-regulated protein 78 (GRP89) [35]. The large number of receptors enables SARS-CoV-2 to infect a broad spectrum of cells, namely alveolar epithelial cells, ciliated cells, olfactory epithelial cells, intestinal cells, endothelial cells, and renal parenchymal cells. After release of the virion from the receptor by the action of transmembrane protease serine subtype 2 (TMPRSS2) and cathepsin L in the endosome, proteins of the viral replicase-transcriptase complex are synthesized by the host. This complex subsequently replicates viral RNA and generates the mRNAs for production of structural and accessory (nucleoprotein, membrane, envelope spike) proteins at the endoplasmic reticulum (ER), which are subsequently integrated into the ER–Golgi intermediate compartment (ERGIC) for virion assembly. The positive sense RNA is incorporated into the newly synthetized virion and the virions are secreted.

Virus load was correlated to severity of ARDS for influenza A and SARS-CoV-2 infections [36,37]. SARS-CoV, MERS-CoV, influenza A virus H1N1 2009, and SARS-CoV-2 viruses induce the hyperinflammation by upregulation of cytokines. This effect, usually termed “cytokine storm”, reflects the loss of homeostasis between pro-inflammatory and anti-inflammatory reaction, and the cytokine pattern is virus-specific. Specific effects are the induction of thick and copious mucus in the airways with potential impairment of mucociliary clearance by SARS-CoV-2 [38] and the inhibition of ENaC fluid transport delaying the resorption of pulmonary edema by influenza A viruses [28]. Prognosis of virus-induced ARDS is also different. The fatality rate of SARS-CoV infections is 9.6%; that of MERS-CoV, 34%; and that of SARS-CoV-2, 5.3% [39]. It was reported that MERS-CoV infections progress more rapidly to ARDS and that DAD was more pronounced in MERS-CoV and SARS-CoV pneumonia than in SARS-CoV-2. The comparison of morbidity and mortality by seasonal influenza A 2018–2019 compared to SARS-CoV-2 showed that the latter had a higher potential for respiratory pathogenicity, leading to more respiratory complications and to higher mortality [40]. Further, age-dependency differs between the viruses. The fatality of patients with SARS-CoV hospitalized in Hong Kong was 13.2% for individuals <60 y and 43.9% for patients >60y [41]. Strong age-dependency of SARS-CoV infections was also seen in another study with mortality rates of 1% (<25 y), 6% (25–44 y), 15% (45–64 y), and 50% (≥65 y) [42]. In contrast, young age was a principal mortality risk factor in the influenza A virus H1N1 2009 pandemic [43].

The higher vulnerability of older lungs can be explained by altered oxidative stress machinery, changes in innate immune system, and increased expression of pro-inflammatory genes in older patients. Morphological and physiological alterations in older compared to younger lungs include the higher fraction of senescent cells with increased advanced glycation end-products (RAGE) expression; dysregulated neutrophil migration, decrease of neutrophil extracellular traps (NET) formation higher myeloperoxidase (MPO), higher macrophage migration inhibiting factor (MIF)-1α, TNF-α, IL-6, and IL-8 levels; compromised endothelial barrier; and increased angiotensin 2 (Ang2) and tissue-type plasminogen activator (tPA) expression. Immune cell function differs in the way that alveolar macrophages show increased expression of genes associated with lung injury and fibrosis and decreased phagocytic capacity. Chemotaxis, phagocytosis, microbial killing, and NET formation are impaired in neutrophils of older individuals. Further, endothelial permeability regulated by Ang2 is higher in older lungs and NADPH oxidase 4 (ROS production) upregulated [7]. Animal data showed higher CD80 and CD86 expression upon lipopolysaccharide (LPS) challenge in older mice and increased MIF-1α levels, whereas antigen presentation and bacterial and viral clearance were decreased. The fact that mortality by the influenza A virus H1N1 was lower in older people may suggest that a less active immune function may potentially prevent hyperinflammation. The formation of fibrosis occurred in about 10% of ARDS caused by influenza A virus H1N1 [44]. Thirty-three percent of MERS-CoV survivors developed lung fibrosis [45]. Two years after SARS-CoV-induced ARDS, 52% of survivors showed indication for lung fibrosis [46]. More than 33% of recovered coronavirus disease (COVID-19) patients showed fibrotic abnormalities on hospital discharge [47] and, after 1 year, 25% of severely ill patients showed an indication of pulmonary fibrosis according to another study [48].

The reason for the observed differences in ARDS is not entirely clear, and there are also other open questions like for instance the role of macrophages in influenza A virus-induced ARDS, because influenza A strains infect macrophages to different extents [29]. In addition, the exact contribution of NETs to pathophysiology is unclear. NETs are fibrous structures that contain neutrophil granular proteins coated on a backbone structure of DNA. High neutrophil counts are predictors for poor outcome, which can be explained by the adverse effects of elastase and MPO. NETs may further obstruct airways; induce inflammation; and trigger immunothrombosis, deposition of fibrin, and, thereby, reduce oxygenation. However, even though NET formation was higher in machine-ventilated patients, the reduction of NETs did not shorten the time of ventilation. In a later phase, the neutrophils appear to participate in the remodeling of the damaged tissue through release of matrix metalloproteinase 9 (MMP-9) and activation of the Wnt/β-catenin pathway, and stimulate proliferation of AT2 cells. Consistent with the reported positive effects of NETs, impaired neutrophil function facilitated the progression of influenza A virus H3N2 pneumonia in mouse models and depletion of neutrophils was linked to more severe illness. It was concluded that neutrophils are involved in the first phase as inducers of epithelial damage but also in the second phase to improve resolution of the disease and are, therefore, no good target for treatment [49].

Hyperinflammatory phenotype has been associated with MODS and mortality, but SARS-CoV-2-related ARDS was associated with lower prevalence of hyperinflammatory syndrome and excess mortality was not linked to hyperinflammatory pathways [50]. There is other uncertainty regarding the relevance of Ang2 levels. On the one hand, increased ACE2 levels of older mechanically ventilated COVID-19 patients compared to older not ventilated individuals suggest that the high ACE2 expression facilitates virus entry [51]. On the other hand, ACE2 levels severely decreased in pulmonary fibrosis, and recombinant ACE2 is considered as a treatment of ARDS and PAH. In influenza A virus H1N1, ACE2 was also downregulated and in avian flu (H5N1 and H7N9) increased Ang2 levels were correlated to worse prognosis [52]. The lack of clear causative relationships hinders the identification of potential drugs for therapy of virus-induced ARDS.

### 2.2. Treatment Options for ARDS

Due to the complex pathology of ARDS, a broad panel of drugs with anti-inflammatory, vasodilatatory, anti-thrombotic, and anti-oxidative action have been evaluated as potential treatment options. Several drugs, such as salbutamol, neutrophil elastase inhibitors, non-steroidal anti-inflammatory drugs (acetylsalicylic acid, ibuprofen), inhaled nitric oxide, and transient receptor potential vanilloid 4 (TRPV4) inhibitors GSK1016790, did not show convincing effects [53,54,55]. Immunomodulatory drugs elafin; alpha-1-antitrypsin; imatinib; bevacizumab; anti-IFN-γ; pirfenidone and tetracycline; agents on channel dysfunction GSK634775, GW328267C, and CGS-21680; RAGE inhibitors; the plasma-free hemoglobulin scavenger haptoglobulin; the anticoagulant antithrombin; and the pro-resolution agent lipoxin A4 showed promising results in animal experiments and need confirmation in clinical trials. More information on this topic is available elsewhere (e.g., [54,55]). Since controversial results have been published, classifying patients into subgroups and patient endotypes and identification of parameters linked to higher risk has been suggested for a better assessment of the drugs. A useful parameter for classification may be disease severity because in mild ARDS; mortality is 27%; in moderate, 35%; and in severe, 45% of patients. The PaO_2_ to FiO_2_ ratio according to the Berlin definition of ARDS defines the severity of the disease and correlates to the mortality rates. Another possibility could be the underlying pathology. It was reported that sepsis-related ARDS had a higher mortality than non-sepsis-related ARDS and that trauma-associated ARDS was less severe than non-trauma-associated. Patient endotypes also exist, and ARDS patients presenting high pro-inflammatory markers, metabolic acidosis, and higher vasopressor requirements profited from simvastatin treatment, while other patients with ARDS did not [56]. It may be expected that ARDS caused by infection with SARS-CoV-2 represent a more homogenous collective than hospitalized ARDS patients prior to December 2019 and that the identification of appropriate treatment may be easier.

The SARS-CoV-2 pandemic caused a great need for efficient treatment of virus-induced ARDS, leading to a prominent increase in the number of clinical trials. There were slightly more than 1000 clinical trials posted in clinicaltrials.gov for the treatment of ARDS in the 40 years (30 November 1979 and 30 November 2019) prior to the outbreak of the COVID-19 pandemic compared to more than 550 studies in the last 22 months (1 December 2019 to 20 September 2021). Compounds in clinical trials, which were neither terminated, completed, suspended, withdrawn, or have unknown status, are listed in Table S1 (Supplementary Material).

Compounds that are evaluated in the current trials to decrease mortality and severity of ARDS include anti-inflammatory therapies, either by corticosteroids (dexamethasone, methylprednisolone) or by non-steroidal anti-inflammatory drugs (acetaminophen, ibuprofen). Other trials study antagonization of IL-6 (siltuximab, olokizumab), IL-6 receptor (tocilizumab, sarilumab and levilimab-completed, no results) and JAK-signaling cascade (ruxolitinib, baricitinib). Further targets are INF-γ (emapalumab, trial terminated due to recruitment issues), IL-1β and IL-1β receptor (anakinra, canakinumab), CCR-5 (cenicriviroc, leronlimab) and tumor necrosis factor (TNF)-α (infliximab), and inhibition of complement C3 (APL-9, AMI-101) or C5 (ravulizumab, Zilucoplan^®^) activation [57]. Although previous clinical trials combating coagulation in ARDS did not produce convincing results [58], a decrease of coagulation in ARDS is the goal of ongoing trials studying heparin, antithrombin III, defibrotide, and tPA. Other strategies focus on the decrease of hypertension by inhibition of Ang2 with statins or administration of non-hypertensive Angiotensin 1–7 [A(1–7)] or vasodilation. In a small study, statin treatment of ARDS patients seemed to improve health in terms of organ failure and also by lowering the need for ventilation [59]. Vasodilation using inhaled nitric oxide improved arterial oxygenation with no effect on survival rate and morbidity of critical patients [60]. It is, however, possible that vasoactive intestinal peptide, ambrisentan, iloprost, and BAY1211163 or BAY1097761, which are evaluated in ongoing clinical trials, will show better effects. Agents with anti-oxidant effects have found to be beneficial in a variety of diseases but most of the antioxidant therapies (N-acetylcysteine, selenium, vitamin C, oxothiazolidine-4-carboxylic acid, lactoferrin, and lisofylline) could not improve the all-cause mortality and might have even been harmful in ARDS patients at low risk of death [61]. Mesenchymal stem cells (MSC) display a variety of positive effects, ranging from a decrease of inflammation and stimulation of regeneration to inhibition of fibrosis [62]. Expectations are high that they will improve acute and long-term effects of ARDS. Clinical trials assess the effects of both entire cells and MSC-derived exosomes. The use of anti-viral therapies (remdesivir, hydroxychloroquine, ganciclovir, and maraviroc) raised great hopes [63], but small trials with lopinavir/ritonavir, favipiravir, and umifenovir did not produce beneficial effects [55]. Other agents under evaluation include vadadustat, VERU-111, dornase alpha, sagramostim, tazemetostat, small polyanions, etc. [55]. Lungs of patients recovering from ARDS often do not recover completely. Since fibrotic changes and tissue alterations similar to PAH are seen in these patients, therapies preventing tissue remodeling may be beneficial. It may be suggested that drugs used for the treatment of PAH, such as ET-1 receptor antagonists, phosphodiesterase 5 inhibitors, guanylate cyclase stimulators, and prostaglandin analogues, may act as beneficial in ARDS [64].

The insufficient treatment options for ARDS may indicate that contribution of potential additional factors is not recognized. Fluid clearance by lymphatic vessels was more reduced in influenza A virus H5N1 than in influenza A virus H1N1 infections, which may explain the higher mortality of H5N1 of 53–60% compared to H1N1 with 1–4%. Damage of lymphatic vessels by infection with SARS-CoV-2 is likely because the endothelia express the ACE2 receptor [65]. In addition, the glycocalyx of lymphatic endothelia is similar to endothelia of blood vessels and may allow attachment of the virus [66]. Since the lymphatics also have a role in the immune system, the authors of that study hypothesized that the phenomenon of the cytokine storm may be caused by disorder of lymphatic flow.

### 2.3. Pulmonary Lymphatic System

The lungs need a well-developed lymphatic system for protection against the high exposure to particles and toxicants from the environment. The vessel network starts with small lymphatic capillaries (also called initial or terminal lymphatics) that gradually combine to form larger diameter vessels, namely the pre-collectors and collectors. The collectors pass into the cortex of the local lymph nodes. After passage of the lymph nodes, trunks (bronchomediastinal trunk for the lungs) and finally the thoracic duct on the left side and the right lymphatic duct are formed [67].

#### 2.3.1. Architecture of the Pulmonary Lymphatic System

**Figure 4:**
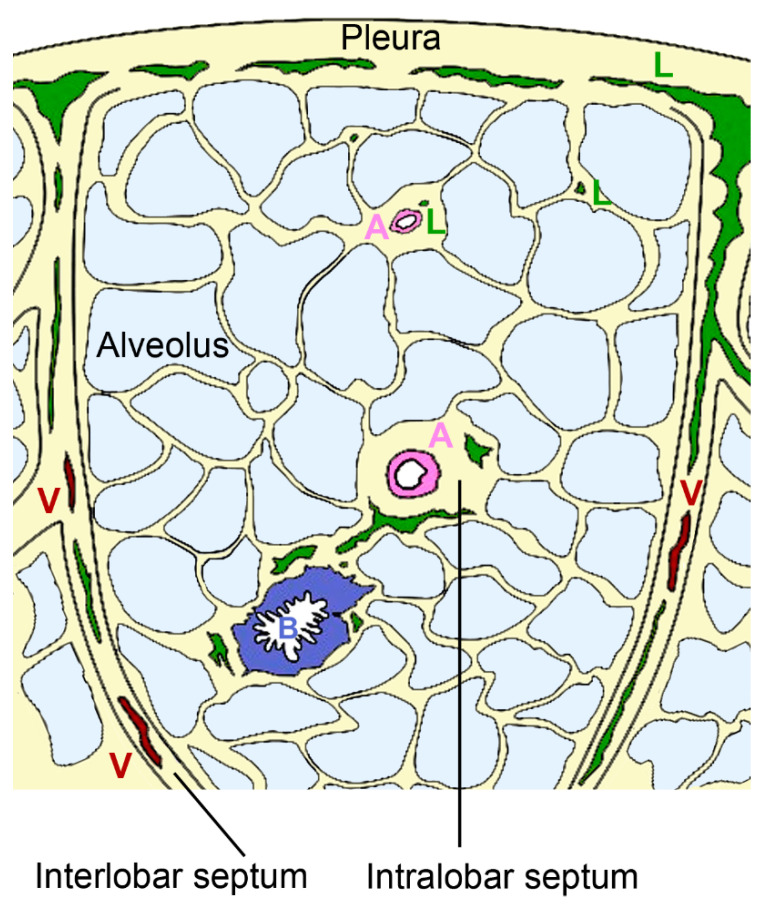
Organization of pulmonary lymphatics. Interlobular lymphatics (L) start at the pleura and follow the veins (V) in the interlobular septa. Lymphatic vessels are also found in bronchovascular bundles that contain bronchus (B) and artery (A) in the perivascular space of arteries of intralobular septa and independent from vessels in interalveolar septa.

Pulmonary lymphatics extend deep into the pulmonary lobules in association with bronchioles and intralobular arterioles or arise in the pleura and accompany the interlobular veins [68] (Figure 4).

The intralobular lymphatic vessels are further subdivided into bronchovascular, perivascular, peribronchiolar, and interalveolar vessels. Most of the intralobular lymphatics are in close contact with blood vessels; a minority is associated with bronchioles (~7%) and occasionally (<1%) small lymphatics are present in interalveolar septa without obvious association to blood vessels or bronchioles [69]. Staining with D2–40 as a marker for lymph vessels revealed that in human lungs 53% of blood vessels with <50 µm diameter were associated with lymphatic capillaries [70]. The association with arteries appears physiologically relevant because both endothelial and smooth muscle cells express vascular endothelial growth factor (VEGF)-C, which acts as growth factor for lymphatic vessels [71]. The coverage with lymphatic vessels in the interalveolar space is sparse and only 3.6–19% of the alveoli are associated to lymphatic structures [72]. This low coverage suggests that these vessels do not play a prominent role in the resorption of pulmonary edema. The small perivascular lymphatics and the few peribronchiolar lymphatics are probably the most important for resorption. They form two systems with opposing fluid direction; lymph from the lung periphery is transported to the pleura, while lymph from other lung regions is transported to the hilum [73]. Lymphatic flow can increase 3–10 times in the setting of hydrostatic pulmonary edema [74]. Hydrostatic pressure defines the force that drives the fluid out of blood vessels, while oncotic pressure is related to macromolecules in the blood and helps to retain fluid in the blood vessels. Finally, also membrane permeability, representing the ease to which fluid passes the vessel wall, defines the extent of pulmonary edema. Lymph flow is influenced by breathing, depth of ventilation, tissue hydrostatic pressure, intrinsic pumping of collecting lymphatics, and systemic venous pressure in the extremities. In the lungs, drainage primarily relies on respiration-associated pressure changes rather than vessel contraction.

#### 2.3.2. Morphology of Lymphatic Vessels

**Figure 5:**
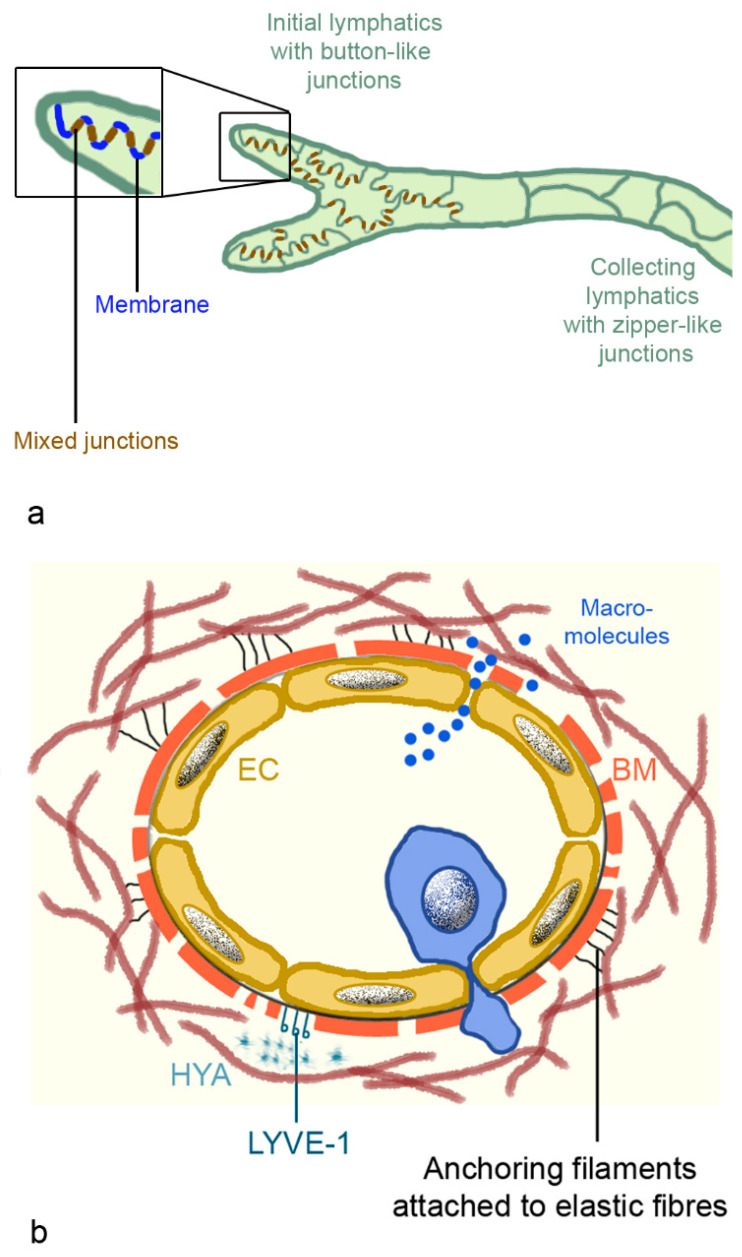
Intercellular junctions of lymphatic capillaries and collecting lymphatics (**a**), and scheme of lymphatic capillaries (**b**). (**a**): Mixed junctions consist of adherent junctions with vascular endothelial (VE) cadherin; β-catenin and p120 catenin; and tight junctions containing claudin 5, occludin, ZO-1, junction adhesion molecule A (JAM-A), and endothelial cell-selective adhesion molecule (ESAM). Membranes between the buttons possess platelet endothelial cell adhesion molecule-1 (PECAM-1) and lymphatic vessel endothelial hyaluron receptor 1 (LYVE-1). (**b**): Lymph capillaries possess an endothelial layer, discontinuous basal membrane, elastic fibers, and anchoring filaments to fix them in the interstitium. Dendritic cells (blue cell) actively migrate into the vessels, while macromolecules can enter the vessel by diffusion. Abbreviation: BM, basal membrane; EC, endothelial cell; HYA, hyaluronan.

The fine structure of lymph capillaries was studied in detail in murine lungs. Epithelia have an oak leaf-shape, which allows interdigitation and loose discontinuous junctions. The alternating membrane flaps are linked at their sides by discrete assemblages (3 µm wide and 3 µm spaced) [75]. The assembly complexes, buttons, and zippers contain the adhesion proteins: vascular epithelial (VE)-cadherin, β-catenin, and p120 catenin, and the tight junction proteins: ZO-1, occludin, endothelial cell-selective adhesion molecule (ESAM), and junction adhesion molecule (JAM)-A. At their tips, the flaps are free and generate openings of 0.5–1 µm, which are decorated with platelet endothelial cell adhesion molecule-1 (PECAM-1, CD31) and lymphatic vessel endothelial hyaluron receptor 1 (LYVE-1) (Figure 5a). There is no continuous basement membrane and the flap-like structures connect directly to the surrounding structures (organs or tissues), via thin, elastic fibers (Figure 5b). The attachment ensures patency of the vessels that are prone to collapse because they lack continuous basement membrane, smooth muscle cells, and pericytes [76]. The capillaries are highly permeable for macromolecules, including antigens like viruses and bacteria and immune cells. Diffusion of macromolecules is passive, and uptake of HYA is done via binding to LYVE-1 [76]. Lymphatics are involved in cell trafficking, which represents more intra- than extravasation. Lymph fluid may contain erythrocytes, monocytes, and dendritic cells, which leaked from the blood vessels into the interstitial spaces. Compared to blood vessels, lymphatic vessels, due to the lack of a continuous basement membrane and the absent/low cell coverage, expose leukocytes to little shear stress [75]. Dendritic cells have to enter actively because they are larger (10–15 µm) than lymphocytes (7–10 µm). The unique architecture of the lymphatic vessels is the compromise between high permeability for fluid and macromolecules, and more restriction for leukocyte entry. Extravasation from blood vessels and intravasation into lymph vessels is increased upon inflammation. At the extremities, pre-collecting lymphatics have one-way valves at more irregular intervals and sparse smooth muscles in their walls. The larger collecting lymphatics possess valves at regular intervals to prevent retrograde backflow and smooth muscles, and are capable to perform contractions. By contrast, pulmonary pre-collecting and collecting lymphatics have valves but lack smooth muscle cells [66].

### 2.4. Lymphatic Vessels in Pulmonary Diseases

#### 2.4.1. Role in Acute Lung Injury/Pulmonary Edema

A functional pulmonary lymphatic system is important for lung development and for expansion of the lungs after birth [72]. Also, later in life, lymphatic function is essential for lung health, which can be easily seen in lung transplants, where the lymphatic vessels were severed and pulmonary edema developed fast [76]. Resorption of pulmonary edema plays a critical role for the outcome of ARDS.

Fluid from the alveolar lumen is transported by ENaC and Na/K-ATPase of the alveolar epithelium from the alveolar lumen to the interstitium. This has to be followed by removal of the fluid from the interstitium. Improved fluid clearance and survival in LPS-induced acute lung damage of rats was linked to increased expression and activity of sodium channel, Na/K-ATPase and LYVE-1, suggesting increased lymphangiogenesis [77]. Fluid in the interstitial spaces contains water bound to HYA and proteins as the main components. HYA is a linear, non-sulfated β-1,4-linked polymer of a repeated disaccharide of (1–3)- and (1–4)-linked β-d-glucuronic acid and N-acetyl β-d-glucosamine monomer with a molecular weight of 10^5^ to 10^7^ Da. The molecule is stabilized by hydrogen bonds, which gives it a double helical configuration and enables the binding of 1000-fold its own size of water [78]. The concentration of HYA in the lymphatics is 0.2–50 mg/L and 10–100 times higher than in plasma [79]. HYA displays tissue-specific effects, either on physiological functions (lubrication, hydration balance, matrix structure, and steric interactions) or on cellular interactions (cell differentiation, proliferation, development, and recognition) in tissues with high HYA levels [80]. In organs with low HYA levels, like the lungs, increased HYA levels may have negative effects. At homeostasis, HYA production through three HYA synthases (HAS1-3) is balanced by cellular uptake and degradation via three hyaluronidases (Hyal1-3).

Intrapulmonary HYA levels were reported to be linked to a variety of human respiratory diseases, e.g., chronic obstructive pulmonary disease (COPD), asthma, idiopathic pulmonary fibrosis (IPF), idiopathic PAH, sarcoidosis, etc. [81]. HYA content was increased in ARDS to 8080% of the normal amount [82], and HYA exudates in the alveolar spaces have been detected in ARDS caused by SARS-CoV-2 [83]. In contrast to healthy lungs, not only the total amount but also the molecular weight and structure of HYA were altered in damaged lungs. It was reported that upon pulmonary damage HYA was degraded to fragments of 70–500 kDa and the low molecular weight HYA propagated the inflammation [84]. This fragmentation may be induced by release of reactive oxygen species from neutrophils or by action of Hyal1 and Hyal2 enzymes from dying cells. Further, HYA was covalently modified by heavy chains from inter-alpha inhibitor by tumor-necrosis-factor-stimulated-gene-6 (TSG-6) [81]. This modification facilitated the binding of leukocytes and promoted inflammation. It has also been proposed that modification by inter-alpha inhibitor may protect HYA from degradation [85].

After the healing of acute pulmonary lesions, HYA levels in BAL, sputum, and tissue decreased. It is possible that HYA may serve as a biomarker for lung damage or as a treatment target. Antenatal administration of betamethasone decreased lung HYA concentration and normalized lung function in preterm rabbit pups [86]. To obtain further insight into this pathogenic mechanism, the relation of O-N-acetylglucosamine glycosylation, low and high molecular weight HYA and HAS2 protein levels in ARDS will be studied in a study starting in October 2021 (NCT05055557).

#### 2.4.2. Role in Chronic Pulmonary Diseases

If HYA is not removed, chronic inflammation and collagen deposition lead to fibrosis and tissue remodeling [84]. In addition to HYA levels, changes in the density of lymphatics have been reported. Diminished lymphangiogenesis has been reported in asthma, whereas severe chronic COPD and IPF are characterized by increased lymphangiogenesis. A role of lymphatics in pulmonary fibrosis was identified in animal models and the pan-tyrosine kinase inhibitor Nintedanib, which is approved for the treatment of IPF, stimulated angiogenesis, and lymphangiogenesis. The density of lymphatic vessels increased in parallel to the fibrotic changes in IPF, suggesting fibrosis-promoting rather than rescuing effects of lymphangiogenesis in lung injury [87]. Other data suggest beneficial effects of lymphoangiogenesis in damaged lungs. In patients with lung fibrosis and organizing pneumonia, severe lymphatic vessel damage was seen. In pneumonia without resulting fibrosis, active lymphangiogenesis occurred in the alveolar lesions [88]. VEGF-C, which promotes lymphangiogenesis in the skin, was decreased in bronchoalveolar lavage fluid (BALF) of IPF patients compared to healthy volunteers, and lung fibrosis in the bleomycin-induced mouse model was reduced when lymphangiogenesis was induced by overexpression of VEGF-C [89]. Expansion of the lymphatic network reduced pulmonary macrophage accumulation and increased fluid clearance. It has been hypothesized that the role of lymphatic vessels is linked to the extent of tissue remodeling and the stage of fibrosis. The increased pulmonary lymphangiogenesis in severe COPD was seen in alveolar parenchyma and around bronchioles [90]. These are regions, where in the healthy condition only a few lymphatic vessels can be found, and it is possible that lymphangiogenesis should compensate insufficient functionality of existing lymphatic vessels. It is, on the other hand, not clear, if the newly formed vessels are functional. Coverage of the vessel wall with cells and excess basement membrane material accumulation may lead to insufficient function of lymphatic vessels in lung diseases because uptake of macromolecules and cells is hindered [91]. Such morphological alterations have been detected in bleomycin-induced pulmonary fibrosis of mice, and these changes promoted the accumulation of protein and fibroblasts in the perilymphatic space. The density of lymphatic vessels in lung fibrosis was higher than that of controls, and density in fibrotic areas was higher than in regions of tissue remodeling. This supports an earlier study that concluded that lymphatic vessels contribute to fibrosis maturation and scar formation through the drainage of excessive proteins and fluid during fibrosis [91]. According to this theory, the link of increased lymphatic vessels density in fibrotic areas is not due to a causative role of the lymphatic vessels in lung fibrosis but represents a mechanism to decrease fibrosis.

The contribution of lymphatic vessels in the pathology of ARDS most likely will not result in new pharmacological treatment options because lymphedema of the extremities is mainly treated with manual decompression. Surgical interventions may be beneficial in specific cases. Pharmacological therapy comprises agents that modulate tissue inflammation, fibrosis, and lymphangiogenesis [92]. Utilization of lymphangiogenic growth factors, such as VEGF-C, could be efficient if this growth factor had positive effects not only on the number but also on the function of lymphatic vessels. However, this has not been clearly demonstrated. Ketoprofen and selenium were effective for the treatment of secondary lymphoma of the extremities. Ketoprofen acted through an increase of TNF-α expression with a subsequent increase in VEGF-C. The mode of action of selenium is assumed to be mediated by immunomodulation. Available evidence suggests that various herbs are useful in alleviating lymphedema, but conclusive data showing superiority in the benefit/risk ratio of these products are missing [93].

## 3. Conclusions

The pathology of ARDS is still not completely understood, and additional factors like dysfunction of lymphatic vessels may contribute to the variable outcome of the syndrome and long-term sequelae of ARDS. The mechanism and pathogenic role of HYA in ARDS will be studied in an upcoming trial (NCT05055557), which potentially will result in new treatment modalities. Options to improve the function of pulmonary lymphatics by pharmacological treatment are limited because lymphedema is not very responsive to drugs. Compounds with beneficial action in secondary lymphedema, selenium, or ketoprofen may be suggested as supportive treatment in ARDS, as well as stimulation of lymphangiogenesis by administration of VEDGF-C to prevent fibrosis.

## Supplementary Materials

The following are available online at https://www.mdpi.com/article/10.3390/biomedicines9111732/s1, Table S1: Published not yet completed clinical trials on ARDS

## References

[1] Force A.D.T., Ranieri V.M., Rubenfeld G.D., Thompson B.T., Ferguson N.D., Caldwell E., Fan E., Camporota L., Slutsky A.S. (2012). Acute Respiratory Distress Syndrome: The Berlin Definition. JAMA.

[2] De Luca D., Cogo P., Kneyber M.C., Biban P., Semple M.G., Perez-Gil J., Conti G., Tissieres P., Rimensberger P.C. (2021). Surfactant Therapies for Pediatric and Neonatal ARDS: ESPNIC Expert Consensus Opinion for Future Research Steps. Crit. Care.

[3] Pramanik A., Rosenkrantz T. (2020). Respiratory Distress Syndrome Treatment & Management. Respiratory Distress Syndrome.

[4] Reilly J.P., Calfee C.S., Christie J. (2019). Acute Respiratory Distress Syndrome Phenotypes. Semin. Respir. Crit. Care Med..

[5] Bellani G., Laffey J.G., Pham T., Fan E., Brochard L., Esteban A., Gattinoni L., Van Haren F., Larsson A., McAuley D.F. (2016). Epidemiology, Patterns of Care, and Mortality for Patients with Acute Respiratory Distress Syndrome in Intensive Care Units in 50 Countries. JAMA.

[6] Siegel M., Parsons P. (2020). Acute Respiratory Distress Syndrome: Epidemiology, Pathophysiology, Pathology, and Etiology in Adults.

[7] Brown R., McKelvey M.C., Ryan S., Creane S., Linden D., Kidney J.C., McAuley D.F., Taggart C.C., Weldon S. (2020). The Impact of Aging in Acute Respiratory Distress Syndrome: A Clinical and Mechanistic Overview. Front. Med..

[8] Gibson P.G., Qin L., Puah S.H. (2020). COVID-19 Acute Respiratory Distress Syndrome (ARDS): Clinical Features and Differences From Typical Pre-COVID-19 ARDS. Med. J. Aust..

[9] Absolute Reports (2020). Acute Respiratory Distress Syndrome (ARDS) Market Insight, Epidemiology and Market Forecast -2030. DelveInsight.

[10] Zambon M., Vincent J.-L. (2008). Mortality Rates for Patients with Acute Lung Injury/ARDS Have Decreased over Time. Chest.

[11] Griffiths M.J.D., McAuley D.F., Perkins G.D., Barrett N., Blackwood B., Boyle A., Chee N., Connolly B., Dark P., Finney S. (2019). Guidelines on the Management of Acute Respiratory Distress Syndrome. BMJ Open Respir. Res..

[12] Gehr P., Bachofen M., Weibel E.R. (1978). The Normal Human Lung: Ultrastructure and Morphometric Estimation of Diffusion Capacity. Respir. Physiol..

[13] Rehfeld A., Nylander M., Karnov K., Rehfeld A., Nylander M., Karnov K. (2017). The Respiratory System. Compendium of Histology.

[14] MacLaren R., Stringer K.A. (2007). Emerging Role of Anticoagulants and Fibrinolytics in the Treatment of Acute Respiratory Distress Syndrome. Pharmacother. J. Hum. Pharmacol. Drug Ther..

[15] Bongard F.S., Matthay M., Mackersie R.C., Lewis F.R. (1984). Morphologic and Physiologic Correlates of Increased Extravascular Lung Water. Surgery.

[16] Jacobson J., Garcia J., Mason R., Broaddus V., Martin T., King T., Schraufnagel D., Murray J., Nadel J. (2010). Pulmonary Circulation and Regulation of Fluid Balance. Murray and Nadel’s Textbook of Respiratory Medicine.

[17] Aberle D.R., Wiener-Kronish J.P., Webb W.R., Matthay A.M. (1988). Hydrostatic versus Increased Permeability Pulmonary Edema: Diagnosis Based on Radiographic Criteria in Critically Ill Patients. Radiology.

[18] Cheung O., Graziano P., Smith M., Leslie K., Wick M. (2018). Acute Lung Injury. In Practical Pulmonary Pathology: A Diagnostic Approach.

[19] Revercomb L., Hanmandlu A., Wareing N., Akkanti B., Karmouty-Quintana H. (2021). Mechanisms of Pulmonary Hypertension in Acute Respiratory Distress Syndrome (ARDS). Front. Mol. Biosci..

[20] Beiderlinden M., Kuehl H., Boes T., Peters J. (2006). Prevalence of Pulmonary Hypertension Associated with Severe Acute Respiratory Distress Syndrome: Predictive Value of Computed Tomography. Intensiv. Care Med..

[21] Ñamendys-Silva S., Santos-Martínez L., Pulido T., Rivero-Sigarroa E., Baltazar-Torres J.A., Dominguez-Cherit G., Sandoval J. (2014). Pulmonary Hypertension Due to Acute Respiratory Distress Syndrome. Braz. J. Med Biol. Res..

[22] Sadigov A., Akhundov S.S., Agayeva A. (2021). Post-Acute Respiratory Distress Syndrome Pulmonary Fibrosis and Pulmonary Artery Hypertension in Patients Affected by Severe COVID19. Am. J. Respir. Crit. Care Med..

[23] Matthay M.A., Zemans R.L., Zimmerman G.A., Arabi Y., Beitler J.R., Mercat A., Herridge M., Randolph A.G., Calfee C.S. (2019). Acute Respiratory Distress Syndrome. Nat. Rev. Dis. Prim..

[24] Smith J.S., Perez R., Gorbett D., Mueller J., Daniels C.J. (2013). Pulmonary Hypertension Idiopathic Pulmonary Fibrosis: A Dastardly Duo. Am. J. Med Sci..

[25] Thille A.W., Esteban A., Fernández-Segoviano P., Rodriguez J.-M., Aramburu J.-A., Vargas-Errázuriz P., Martín-Pellicer A., Lorente A.J., Frutos-Vivar F. (2013). Chronology of Histological Lesions in Acute Respiratory Distress Syndrome with Diffuse Alveolar Damage: A Prospective Cohort Study of Clinical Autopsies. Lancet Respir. Med..

[26] Del Sorbo L., Slutsky A.S. (2011). Acute Respiratory Distress Syndrome and Multiple Organ Failure. Curr. Opin. Crit. Care.

[27] Chang J.C. (2019). Acute Respiratory Distress Syndrome as an Organ Phenotype of Vascular Microthrombotic Disease: Based on Hemostatic Theory and Endothelial Molecular Pathogenesis. Clin. Appl. Thromb..

[28] Brand J.D., Lazrak A., Trombley J.E., Shei R.-J., Adewale A.T., Tipper J.L., Yu Z., Ashtekar A.R., Rowe S.M., Matalon S. (2018). Influenza-Mediated Reduction of Lung Epithelial Ion Channel Activity Leads to Dysregulated Pulmonary Fluid Homeostasis. JCI Insight.

[29] Luyt C., Combes A., Trouillet J.-L., Nieszkowska A., Chastre J. (2011). Virus-Induced Acute Respiratory Distress Syndrome: Epidemiology. Manag. Outcome.

[30] Clementi N., Ghosh S., De Santis M., Castelli M., Criscuolo E., Zanoni I., Clementi M., Mancini N. (2021). Viral Respiratory Pathogens and Lung Injury. Clin. Microbiol. Rev..

[31] Archer S.L., Sharp W.W., Weir E.K. (2020). Differentiating COVID-19 Pneumonia from Acute Respiratory Distress Syndrome and High Altitude Pulmonary: EdemaTherapeutic Implications. Circulation.

[32] Herold S., Becker C., Ridge K.M., Budinger G.S. (2015). Influenza Virus-Induced Lung Injury: Pathogenesis and Implications for Treatment. Eur. Respir. J..

[33] Flerlage T., Boyd D.F., Meliopoulos V., Thomas P.G., Schultz-Cherry S. (2021). Influenza Virus and SARS-CoV-2: Pathogenesis and Host Responses in the Respiratory Tract. Nat. Rev. Genet..

[34] Fenizia C., Galbiati S., Vanetti C., Vago R., Clerici M., Tacchetti C., Daniele T. (2021). SARS-CoV-2 Entry: At the Crossroads of CD147 and ACE2. Cells.

[35] Gadanec L., McSweeney K., Qaradakhi T., Ali B., Zulli A., Apostolopoulos V. (2021). Can SARS-CoV-2 Virus Use Multiple Receptors to Enter Host Cells?. Int. J. Mol. Sci..

[36] Pronier C., Gacouin A., Lagathu G., Le Tulzo Y., Tadié J.-M., Thibault V. (2021). Respiratory Influenza Viral Load as a Marker of Poor Prognosis in Patients with Severe Symptoms. J. Clin. Virol..

[37] Blot M., Jacquier M., Manoha C., Piroth L., Charles P.-E., Glele L.-S.A., Beltramo G., Nguyen M., Bonniaud P., Prin S. (2020). Alveolar SARS-CoV-2 Viral Load Is Tightly Correlated with Severity in COVID-19 ARDS. Clin. Infect. Dis..

[38] Brosnahan S.B., Jonkman A.H., Kugler M.C., Munger J., Kaufman D.A. (2020). COVID-19 and Respiratory System Disorders. Arter. Thromb. Vasc. Biol..

[39] Quan C., Li C., Ma H., Li Y., Zhang H. (2020). Immunopathogenesis of Coronavirus-Induced Acute Respiratory Distress Syndrome (ARDS): Potential Infection-Associated Hemophagocytic Lymphohistiocytosis. Clin. Microbiol. Rev..

[40] Piroth L., Cottenet J., Mariet A.-S., Bonniaud P., Blot M., Tubert-Bitter P., Quantin C. (2021). Comparison of the Characteristics, Morbidity, and Mortality of COVID-19 and Seasonal Influenza: A Nationwide, Population-Based Retrospective Cohort Study. Lancet Respir. Med..

[41] Donnelly A.C., Ghani A., Leung G., Hedley A.J., Fraser C., Riley S., Abu-Raddad L., Ho L.-M., Thach T.Q., Chau P. (2003). Epidemiological Determinants of Spread of Causal Agent of Severe Acute Respiratory Syndrome in Hong Kong. Lancet.

[42] World Health Organization (2003). Consensus Document on the Epidemiology of Severe Acute Respiratory Syndrome (SARS).

[43] Lemaitre M., Carrat F. (2010). Comparative Age Distribution of Influenza Morbidity and Mortality During Seasonal Influenza Epidemics and the 2009 H1N1 Pandemic. BMC Infect. Dis..

[44] Mineo G., Ciccarese F., Modolon C., Landini M.P., Valentino M., Zompatori M. (2011). Post-ARDS Pulmonary Fibrosis in Patients with H1N1 Pneumonia: Role of Follow-Up CT. La Radiol. Med..

[45] Das K.M., Lee E.Y., Singh R., A. Enani M., Al Dossari K., Van Gorkom K., Larsson S.G., Langer R.D. (2017). Follow-Up Chest Radiographic Findings in Patients with MERS-CoV after Recovery. Indian J. Radiol. Imaging.

[46] Ngai J.C., Ko F.W., Ng S.S., To K.-W., Tong M., Hui D.S. (2010). The Long-Term Impact of Severe Acute Respiratory Syndrome on Pulmonary Function, Exercise Capacity and Health Status. Respirology.

[47] Vasarmidi E., Tsitoura E., Spandidos D.A., Tzanakis N., Antoniou K.M. (2020). Pulmonary Fibrosis in the Aftermath of the Covid-19 Era (Review). Exp. Ther. Med..

[48] Wu X., Liu X., Zhou Y., Yu H., Li R., Zhan Q., Ni F., Fang S., Lu Y., Ding X. (2021). 3-Month, 6-Month, 9-Month, and 12-Month Respiratory Outcomes in Patients Following COVID-19-Related Hospitalisation: A Prospective Study. Lancet Respir. Med..

[49] Yang S.-C., Tsai Y.-F., Pan Y.-L., Hwang T.-L. (2020). Understanding the Role of Neutrophils in Acute Respiratory Distress Syndrome. Biomed. J..

[50] Sinha P., Calfee C.S., Cherian S., Brealey D., Cutler S., King C., Killick C., Richards O., Cheema Y., Bailey C. (2020). Prevalence of Phenotypes of Acute Respiratory Distress Syndrome in Critically Ill Patients with COVID-19: A Prospective Observational Study. Lancet Respir. Med..

[51] Baker S.A., Kwok S., Berry G.J., Montine T.J. (2021). Angiotensin-Converting Enzyme 2 (ACE2) Expression Increases with Age in Patients Requiring Mechanical Ventilation. PLoS ONE.

[52] Ni W., Yang X., Yang D., Bao J., Li R., Xiao Y., Hou C., Wang H., Liu J., Yang D. (2020). Role of Angiotensin-Converting Enzyme 2 (ACE2) in COVID-19. Crit. Care.

[53] Boyle A.J., Mac Sweeney R., McAuley D.F. (2013). Pharmacological Treatments in ARDS; a State-of-the-Art Update. BMC Med..

[54] Harman E., Riley L. (2020). Acute Respiratory Distress Syndrome (ARDS) Medication.

[55] Horie S., McNicholas B., Rezoagli E., Pham T., Curley G., McAuley D., O’Kane C., Nichol A., Dos Santos C., Rocco P.R.M. (2020). Emerging Pharmacological Therapies for ARDS: COVID-19 and Beyond. Intensiv. Care Med..

[56] Kassirian S., Taneja R., Mehta S. (2020). Diagnosis and Management of Acute Respiratory Distress Syndrome in a Time of COVID-19. Diagnostics.

[57] Pum A., Ennemoser M., Adage T., Kungl A.J. (2021). Cytokines and Chemokines in SARS-CoV-2 Infections—Therapeutic Strategies Targeting Cytokine Storm. Biomolecules.

[58] Cornet A., Oudemans-van Straaten H., Schultz M., Juffermans N., Tuinman P. (2014). Anticoagulants for ARDS: Facts and Future. Neth. J. Crit. Care.

[59] Feng Y. (2018). Efficacy of Statin Therapy in Patients with Acute Respiratory Distress Syndrome/Acute Lung Injury: A Systematic Review and Meta-Analysis. Eur. Rev. Med Pharmacol. Sci..

[60] Monsalve-Naharro J., Domingo-Chiva E., García Castillo S., Cuesta-Montero P., Jiménez-Vizuete J. (2017). Inhaled Nitric Oxide in Adult Patients with Acute Respiratory Distress Syndrome. Farm. Hosp..

[61] Bo L., Jin F., Ma Z., Li C. (2021). Redox Signaling and Antioxidant Therapies in Acute Respiratory Distress Syndrome: A Systematic Review and Meta-Analysis. Expert Rev. Respir. Med..

[62] Fröhlich E. (2021). Therapeutic Potential of Mesenchymal Stem Cells and Their Products in Lung Diseases—Intravenous Administration versus Inhalation. Pharmaceutics.

[63] Cao B., Wang Y., Wen D., Liu W., Wang J., Fan G., Ruan L., Song B., Cai Y., Wei M. (2020). A Trial of Lopinavir–Ritonavir in Adults Hospitalized with Severe COVID-19. N. Engl. J. Med..

[64] Duarte J.D., Hanson R.L., Machado R.F. (2013). Pharmacologic Treatments for Pulmonary Hypertension: Exploring Pharmacogenomics. Futur. Cardiol..

[65] Li J., Gao J., Xu Y.-P., Zhou T.-L., Jin Y.-Y., Lou J.-N. (2007). Expression of Severe Acute Respiratory Syndrome Coronavirus Receptors, ACE2 and CD209L in Different Organ Derived Microvascular Endothelial Cells. Zhonghua yi xue za zhi.

[66] Wu C., Li H., Zhang P., Tian C., Luo J., Zhang W., Bhandari S., Jin S., Hao Y. (2020). Lymphatic Flow: A Potential Target in Sepsis-Associated Acute Lung Injury. J. Inflamm. Res..

[67] Margaris K., Black R.A. (2012). Modelling the Lymphatic System: Challenges and Opportunities. J. R. Soc. Interface.

[68] Weber E., Sozio F., Borghini A., Sestini P., Renzoni E. (2018). Pulmonary Lymphatic Vessel Morphology: A Review. Ann. Anat. Anat. Anz..

[69] Sozio F., Rossi A., Weber E., Abraham D.J., Nicholson A.G., Wells A.U., Renzoni E.A., Sestini P. (2012). Morphometric Analysis of Intralobular, Interlobular and Pleural Lymphatics in Normal Human Lung. J. Anat..

[70] Kambouchner M., Bernaudin J.-F. (2009). Intralobular Pulmonary Lymphatic Distribution in Normal Human Lung Using D2-40 Antipodoplanin Immunostaining. J. Histochem. Cytochem..

[71] Partanen T.A., Arola J., Saaristo A., Jussila L., Ora A., Miettinen M., Stacker S.A., Achen M.G., Alitalo K. (2000). VEGF-C and VEGF-D Expression in Neuroendocrine Cells and their Receptor, VEGFR-3, in Fenestrated Blood Vessels in Human Tissues. FASEB J..

[72] Stump B., Cui Y., Kidambi P., LaMattina A.M., El-Chemaly S. (2017). Lymphatic Changes in Respiratory Diseases: More than Just Remodeling of the Lung?. Am. J. Respir. Cell Mol. Biol..

[73] Schraufnagel D.E. (2010). Lung Lymphatic Anatomy and Correlates. Pathophysiology.

[74] Barile M. (2020). Pulmonary Edema: A Pictorial Review of Imaging Manifestations and Current Understanding of Mechanisms of Disease. Eur. J. Radiol. Open.

[75] Jackson D.G. (2019). Leucocyte Trafficking via the Lymphatic Vasculature—Mechanisms and Consequences. Front. Immunol..

[76] Cui Y., Liu K., LaMattina A.M., Visner G., El-Chemaly S. (2017). Lymphatic Vessels: The Next Frontier in Lung Transplant. Ann. Am. Thorac. Soc..

[77] Zhang P., Han J., Cao F., Liu Y., Tian C., Wu C., Smith F.G., Hao Y., Jin S. (2020). PCTR1 improves Pulmonary Edema Fluid Clearance Through Activating the Sodium Channel and Lymphatic Drainage in Lipopolysaccharide-Induced ARDS. J. Cell. Physiol..

[78] Laurent T.C., Fraser J.R. (1992). Hyaluronan. Faseb J..

[79] Tengblad A., Laurent U.B.G., Lilja K., Cahill R.N.P., Engström-Laurent A., Fraser J.R.R., E Hansson H., Laurent T.C. (1986). Concentration and Relative Molecular Mass of Hyaluronate in Lymph and Blood. Biochem. J..

[80] Gupta R.C., Lall R., Srivastava A., Sinha A. (2019). Hyaluronic Acid: Molecular Mechanisms and Therapeutic Trajectory. Front. Veter- Sci..

[81] Lauer M.E., Dweik R.A., Garantziotis S., Aronica M.A. (2015). The Rise and Fall of Hyaluronan in Respiratory Diseases. Int. J. Cell Biol..

[82] Modig J., Hällgren R. (1989). Increased Hyaluronic Acid Production in Lung—A Possible Important Factor in Interstitial and Alveolar Edema during General Anesthesia and in Adult Respiratory Distress Syndrome. Resuscitation.

[83] Hellman U., Karlsson M.G., Engström-Laurent A., Cajander S., Dorofte L., Ahlm C., Laurent C., Blomberg A. (2020). Presence of Hyaluronan in Lung Alveoli in Severe Covid-19: An Opening for New Treatment Options?. J. Biol. Chem..

[84] Lee-Sayer S.S.M., Dong Y., Arif A.A., Olsson M., Brown K.L., Johnson P. (2015). The Where, When, How, and Why of Hyaluronan Binding by Immune Cells. Front. Immunol..

[85] Fries E., Kaczmarczyk A. (2003). Inter-Alpha-Inhibitor, Hyaluronan and Inflammation. Acta Biochim. Pol..

[86] Johnsson H., Eriksson L., Sedin G. (2001). Antenatal Betamethasone Administration Decreases the Lung Hyaluronan Concentration in Preterm Rabbit Pups. Pediatr. Res..

[87] El-Chemaly S., Pacheco-Rodriguez G., Ikeda Y., Malide D., Moss J. (2009). Lymphatics in Idiopathic Pulmonary Fibrosis: New Insights into an Old Disease. Lymphat. Res. Biol..

[88] Ebina M., Shibata N., Ohta H., Hisata S., Tamada T., Ono M., Okaya K., Kondo T., Nukiwa T. (2010). The Disappearance of Subpleural and Interlobular Lymphatics in Idiopathic Pulmonary Fibrosis. Lymphat. Res. Biol..

[89] Baluk P., Naikawadi R.P., Kim S., Rodriguez F., Choi D., Hong Y.-K., Wolters P.J., McDonald D.M. (2020). Lymphatic Proliferation Ameliorates Pulmonary Fibrosis after Lung Injury. Am. J. Pathol..

[90] Mori M., Andersson C.K., Graham G.J., Löfdahl C.-G., Erjefält J.S. (2013). Increased Number and Altered Phenotype of Lymphatic Vessels in Peripheral Lung Compartments of Patients with COPD. Respir. Res..

[91] Meinecke A.-K., Nagy N., Lago G.D., Kirmse S., Klose R., Schrödter K., Zimmermann A., Helfrich I., Rundqvist H., Theegarten D. (2012). Aberrant Mural Cell Recruitment to Lymphatic Vessels and Impaired Lymphatic Drainage in a Murine Model of Pulmonary Fibrosis. Blood.

[92] Forte A.J., Boczar D., Huayllani M.T., Lu X., A (2019). McLaughlin, S. Pharmacotherapy Agents in Lymphedema Treatment: A Systematic Review. Cureus.

[93] Sheikhi-Mobarakeh Z., Yarmohammadi H., Mokhatri-Hesari P., Fahimi S., Montazeri A., Heydarirad G. (2020). Herbs as Old Potential Treatments for Lymphedema Management: A Systematic Review. Complement. Ther. Med..

