# Supplementary material for: Acute Respiratory Distress Syndrome: Focus on Viral Origin and Role of Pulmonary Lymphatics
Source: Biomedicines. 2021 Nov 20;9(11):1732. doi: 10.3390/biomedicines9111732 (PMC8615541; doi:10.3390/biomedicines9111732)
Supplement: Supplementary file 1 [file biomedicines-09-01732-s001.zip › biomedicines-1422412-supplementary.pdf]

Table S1: Overview of trials published on the clinicaltrials.gov website for acute respiratory distress syndrome (ARDS), which were not indicated as completed, terminated or withdrawn by September, 25th 2021

NCT Number	Title	Mechanism	Interventions	Start date
**NCT05035524**	A Randomized Controlled Trial to Investigate The Role of Adjuvant Inhalable Sodium Bicarbonate Solution 8.4% in Treatment of COVID-19	Treatment for severe metabolic acidosis	• Drug: Sodium Bicarbonate Solution	September 1, 2021
**NCT05027815**	Tregs for the Treatment of Acute Respiratory Distress Syndrome (ARDS) Associated With COVID-19 (regARDS)	Regulatory T cells	• Biological: Cryopreserved Ex Vivo Expanded Polyclonal CD4+CD127lo/-CD25+ T Regulatory Cells	August 1, 2021
**NCT05018975**	Tazemetostat for the Treatment of Moderate to Severe COVID-19 Infection	Inhibitor of enhancer of zeste homolog 2 (EZH2), histone-lysine N-methyltransferase enzyme involved in histone methylation	• Drug: Tazemetostat	September 22, 2021
**NCT05000671**	A Study to Evaluate the Safety and Effect of STC314 Injection Continuous Infusion in Subjects With Acute Respiratory Distress Syndrome	Small polyanions to neutralize NET-bound and free histones	• Drug: STC314 injection	August 1, 2021
**NCT04979884**	Safety and Effectiveness of Cyclosporin in the Management of COVID19 ARDS Patients in Alexandria University Hospital	Immunosuppressive drug	• Drug: cyclosporine	August 1, 2021
**NCT04953052**	A Randomized Study to Investigate the Effect of Intravenous Imatinib on the Amount of Oxygen in the Lungs and Blood of Adults With COVID-19 Needing Mechanical Ventilation and Supportive Care.	Protein-tyrosine kinase inhibitor with anti-proliferative and pro-apoptotic properties	• Drug: Imatinib Mesylate	August 2021
**NCT04922957**	A Phase 2b Multi-Center, Randomized, Double-Blind, Placebo-Controlled Study, Evaluating Efficacy and Safety of Allocetra-OTS in Patients With Severe or Critical COVID-19 With Associated Acute Respiratory Distress Syndrome (ARDS)	Early apoptotic cells) shown to have a beneficial effect on cytokine storms	• Drug: ALLOCETRA-OTS	September 2021
**NCT04909697**	Treatment of ARDS With Sivelestat Sodium	Inhibitor of Human Neutrophil Elastase	• Drug: Sivelestat sodium	July 1, 2021
**NCT04905836**	Study of Allogeneic Adipose-Derived Mesenchymal Stem Cells for Treatment of COVID-19 Acute Respiratory Distress	Mesenchymal stem cells	• Biological: COVI-MSC	August 1, 2021

**NCT04903327**	Study of Intravenous COVI-MSC for Treatment of COVID-19-Induced Acute Respiratory Distress	Mesenchymal stem cells	• Biological: COVI-MSC	July 2021
**NCT04836780**	DEXamethasone EARLY Administration in Hospitalized Patients With Covid-19 Pneumonia	Corticosteroid	• Drug: Dexamethasone	June 10, 2021
**NCT04798716**	The Use of Exosomes for the Treatment of Acute Respiratory Distress Syndrome or Novel Coronavirus Pneumonia Caused by COVID-19	Exosomes	• Drug: MSC-exosomes delivered intravenously, different protocols	August, 2021
**NCT04778059**	Safety and Efficacy of USB002 for Respiratory Distress Due to COVID-19	Non-hypertensive Angiotensin 1-7 [A(1-7)]	• Drug: USB002	July 27, 2021
**NCT04588441**	The ARCTIC Trial: Aerosolized Inhaled Adenosine Treatment in Patients With Acute Respiratory Distress Syndrome (ARDS) Caused by COVID-19	Regulator of inflammation	• Drug: Adenosine	September 2021
**NCT04452097**	Use of hUC-MSC Product (BX-U001) for the Treatment of COVID-19 With ARDS	Mesenchymal stem cells	• Biological: Human umbilical cord mesenchymal stem cells	July 1, 2021
**NCT04412395**	Clinical Assessment of Oral Lactoferrin as a Safe Antiviral and Immunoregulatory in Treating COVID-19 Disease	Agent with antiviral and anti-inflammatory effects	• Dietary Supplement: Lactoferrin (Apolactoferrin)	October 1, 2021
**NCT04404387**	Lessening Organ Dysfunction With VITamin C in Septic ARDS	Agent with anti-oxidant properties	• Drug: Administration of vitamin C	September 2021
**NCT04395456**	A Study of the C3 Inhibitor AMY-101 in Patients With ARDS Due to COVID-19 (SAVE)	Complement C3 inhibitor	• Drug: AMY-101	September 2021
**NCT04378244**	CORONA: A Study Using DeltaRex-G Gene Therapy for Symptomatic COVID-19	Retrovector encoding a cytocidal dominant negative human cyclin G1 as genetic payload	• Drug: DeltaRex-G	December 12, 2021
**NCT04377334**	Mesenchymal Stem Cells (MSCs) in Inflammation-Resolution Programs of Coronavirus Disease 2019 (COVID-19) Induced Acute Respiratory Distress Syndrome (ARDS)	Mesenchymal stem cells	• Biological: MSC	September 2021
**NCT04347967**	Mesenchymal Stem Cells for The Treatment of Acute Respiratory Distress Syndrome (ARDS)	Mesenchymal stem cells	• Biological: UMC119-06	September 2021
**NCT04291508**	Acetaminophen and Ascorbate in Sepsis: Targeted Therapy to Enhance Recovery	Non-steroidal anti-inflammatory drug; Agent with anti-oxidant properties	• Drug: Intravenous Acetaminophen<br>• Drug: Intravenous Vitamin C<br>• Drug: 5% Dextrose	September 2021
**NCT04199364**	Medium vs Low Oxygen Threshold for the Surfactant Administration	Pulmonary surfactant	• Drug: Poractant Alfa 80 mg/mL Intratracheal Suspension	June 1, 2021

**NCT04969991**	Study of Varespladib in Patients Hospitalized With Severe COVID-19	Phospholipidase 2 inhibitor with anti-inflammatory effects	• Drug: Varespladib	June 30, 2021
**NCT04843761**	ACTIV-3b: Therapeutics for Severely Ill Inpatients With COVID-19	Nucleoside analog and inhibits the RNA-dependent RNA polymerase; Synthetic vasoactive intestinal polypeptide; Corticosteroid	• Biological: Remdesivir • Biological: Aviptadil• Drug: Corticosteroid	April 20, 2021
**NCT04842747**	VERU-111 in the Treatment of SARS-Cov-2 Infection by Assessing Its Effect on the Proportion of Patients Who Die on Study	Anti-tubulin crosslinks a and b tubulin and inhibits microtubule polymerization	• Drug: VERU-111	May 18, 2021
**NCT04794088**	Intravenous Imatinib in Mechanically Ventilated COVID-19 Patients	Protein-tyrosine kinase inhibitor with anti-proliferative and pro-apoptotic properties	• Drug: Imatinib Mesylate intravenous solution	March 14, 2021
**NCT04710329**	High-Dose Vitamin C Treatment in Critically Ill COVID-19 Patients	Anti-oxidant effect	• Drug: Ascorbic acid	January 16, 2021
**NCT04706507**	Ganciclovir to Prevent Reactivation of Cytomegalovirus in Patients With Acute Respiratory Failure and Sepsis	Competitive inhibitor of deoxyguanosine triphosphate (dGTP) incorporation into DNA	• Drug: IV Ganciclovir	June 29, 2021
**NCT04429555**	Efficacy, Safety, Tolerability, and Biomarkers of MN-166 (Ibudilast) in Patients Hospitalized With COVID-19 and at Risk for ARDS	Phosphodiesterase inhibitor and anti-inflammatory drug	• Drug: Ibudilast	January 11, 2021
**NCT04360096**	Inhaled ZYESAMI™ (Aviptadil Acetate) for the Treatment of Severe COVID-19	Synthetic vasoactive intestinal polypeptide	• Drug: ZYESAMI™ (aviptadil acetate)	February 15, 2021
**NCT04979923**	Efficacy of Nebulized Lidocaine, Salbutamol, and Beclomethasone Plus Salbutamol in the Covid-19 Patients With ARDS on Non-invasive Ventilation; Randomized Control Trial	Local anaesthetic through blockade of voltage-gated sodium channels	• Drug: Lidocaine	July 1, 2021
**NCT04954040**	Prevention and Treatment With Hydroxychloroquine + Azithromycin of Acute Respiratory Syndrome Induced by COVID-19	Treatment and prevention of malaria + Macrolide antibiotic which inhibits bacterial protein synthesis	• Drug: Hydroxychloroquine Pill + Azithromycin Pill	February 10, 2021
**NCT04951882**	Application of hUC-MSCs in Treating Acute Lung Injury: a Single Center Prospective Clinical Research	Mesenchymal stem cells	• Biological: human derived umbilical cord derived mesenchymal stem cells	June 9, 2021
**NCT04940676**	Oral Administration or Nasal Feeding of Huzhangxiefei Decoction for Treatment in Sepsis Induced Acute Lung Injury	Composition and mechanisms unknown	• Drug: 10% Huzhangxiefei Decoction	March 10, 2021
**NCT04865107**	Cellular Immuno-Therapy for COVID-19 ARDS Randomized Clinical Trial	Mesenchymal stem cells	• Biological: UC-MSCs	April 27, 2021

**NCT04804943**	Pilot Clinical Study of NOA-001 for ARDS (Acute Respiratory Distress Syndrome)	Extracorporeal blood purification column for simultaneous elimination of cytokines and activated neutrophils	• Device: NOA-001	May 22, 2021
**NCT04750278**	A Phase 2/3, Randomized, Double Blind, Placebo Controlled, Multicenter Study to Evaluate the Efficacy and Safety of FP-025 in Patients With Severe to Critical COVID 19 With Associated Acute Respiratory Distress Syndrome (ARDS)	Non-hydroxamate matrix metalloproteinase 12 inhibitor	• Drug: FP-025 100 mg or 300 mg	April 6, 2021
**NCT04744090**	Roflumilast in ARDS	Phosphodiesterase-4(PDE4)-Inhibitor with anti-inflammatory effects	• Drug: Roflumilast	March 21, 2021
**NCT04607434**	Mechanism of Delayed Neutrophil Apoptosis in Acute Lung Injury	Anti-oxidant effect	• Drug: Ocotillol Type Ginsenoside Derivatives	January 25, 2021
**NCT04502433**	Poractant Alfa - Curosurf and SARS-COV-19 ARDS (Covid-19)	Pulmonary surfactant	• Drug: CUROSURF® (poractant alfa)	January 6, 2021
**NCT04771000**	A Study of Micro Dose Ambrisentan in Hospitalized Patients With Respiratory Insufficiency Due to COVID-19	Type A endothelin receptor antagonist for PAH treatment	• Drug: Ambrisentan	February 8, 2021
**NCT04536350**	Inhaled Aviptadil for the Treatment of COVID-19 in Patients at High Risk for ARDS	Synthetic vasoactive intestinal polypeptide	• Drug: Aviptadil 67μg	May 18, 2021
**NCT04441047**	Universal Anti-Viral Vaccine for Healthy Elderly Adults	Non-genetically manipulated, patented living immune cells with multiple immunodulatory properties	• Drug: AlloStim	July 12, 2021
**NCT04760821**	Prevention of Acute Myocardial Injury by Trimetazidine in Patients Hospitalized for COVID-19	Small molecule compound with anti-ischemic properties	• Drug: Trimetazidine	December 10, 2020
**NCT04742595**	SARS-CoV-2 Specific Cytotoxic T Lymphocytes for the Treatment of COVID-19 in Patients With Cancer	Cytotoxic T cells	• Biological: SARS-CoV-2 Antigen-specific Cytotoxic T-lymphocytes	December 18, 2020
**NCT04661930**	Fenofibrate for Patients With COVID-19 Requiring Hospitalization	Fenofibrate with lipid-lowering properties	• Drug: TriCor® 145mg tablets	December 13, 2020
**NCT04643691**	Losartan and Spironolactone Treatment for ICU Patients With COVID-19 Suffering From ARDS	Angiotensin II receptor blocker	• Drug: Losartan 50 mg and Spironolactone 25 mg pillules oral use	September 11, 2020
**NCT04594668**	Senicapoc in COVID-19 Patients With Severe Respiratory Insufficiency	Gardos, Ca-activated potassium channel, blocker	• Drug: Senicapoc	April 24, 2020

**NCT04582201**	An Experiment to Evaluate the Safety of agenT-797 in COVID-19 Patients With Severe Difficulty Breathing.	Allogenic human unmodified I natural killer T cells from one healthy donor. Natural killer T cells expressing an invariant a, b T cell receptor (iNKTs) with potential immunomodulating and antineoplastic activities.	• Drug: agenT-797	September 21, 2020
**NCT04511923**	Nebulised Heparin to Reduce COVID-19 Induced Acute Lung Injury	Heparin	• Drug: Nebulised heparin	December 23, 2020
**NCT04510662**	Telmisartan in Respiratory Failure Due to COVID-19	Angiotensin II receptor blocker	• Drug: Telmisartan	August 2020
**NCT04475588**	Efficacy and Safety of Itolizumab in COVID-19 Complications	Anti-CD6 humanized IgG1 monoclonal antibody, CD responsible for priming, activation and differentiation of T cells	• Drug: Itolizumab IV infusion	May 1, 2020
**NCT04468971**	REgulatory T Cell infuSion fOr Lung Injury Due to COVID-19 PnEumonia	Allogenic umbilical cord blood derived regulatory T-cells	• Biological: CK0802	September 29, 2020
**NCT04466098**	Multiple Dosing of Mesenchymal Stromal Cells in Patients With ARDS (COVID-19)	Mesenchymal stem cells	• Biological: Mesenchymal stromal cells	July 30, 2020
**NCT04451291**	Study of Decidual Stromal Cells to Treat COVID-19 Respiratory Failure	Stromal stem cells	• Biological: Decidual Stromal Cells (DSC)	September 25, 2020
**NCT04445246**	Inhaled Iloprost for Suspected COVID-19 Respiratory Failure	Prostacyclin PGI2 analogue with vasodilatory action	• Drug: Inhaled ILOPROST	May 23, 2020
**NCT04441385**	Study to Evaluate the Efficacy and Safety of Maraviroc in SARS-CoV-2 Infection (COVID-19).	CCR5 receptor antagonist, inhibitor of HIV entry	• Drug: Maraviroc 300 mg	June 26, 2020
**NCT04426695**	Safety, Tolerability, and Efficacy of Anti-Spike (S) SARS- CoV-2 Monoclonal Antibodies for Hospitalized Adult Patients With COVID-19	Anti-spike SARS-CoV-2 monoclonal antibodies from recovered patients	• Drug: REGN10933+REGN10987	June 10, 2020
**NCT04402060**	A Study of APL-9 in Adults With Mild to Moderate ARDS Due to COVID-19	Second generation complement C3 inhibitor	• Drug: APL-9	May 28, 2020
**NCT04399889**	hCT-MSCs for COVID19 ARDS	Mesenchymal stem cells	• Biological: human cord tissue mesenchymal stromal cells	June 18, 2020
**NCT04397510**	Nebulized Heparin for the Treatment of COVID-19 Induced Lung Injury	Stimulator of thrombin inactivation by antithrombin III	• Drug: Heparin	June 1, 2020
**NCT04390139**	Efficacy and Safety Evaluation of Mesenchymal Stem Cells for the Treatment of Patients With Respiratory Distress Due to COVID-19	Mesenchymal stem cells	• Drug: XCEL-UMC-BETA	May 13, 2020
**NCT04384445**	Zofin (Organicell Flow) for Patients With COVID-19	Mesenchymal stem cells	• Biological: Zofin	September 8, 2020

**NCT04367077**	MultiStem Administration for COVID-19 Induced ARDS (MACoVIA)	Mesenchymal stem cells	• Biological: MultiStem	April 28, 2020
**NCT04359290**	Ruxolitinib for Treatment of Covid-19 Induced Lung Injury ARDS	JAK1 and JAK2 inhibitor, TK inhibitor	• Drug: Ruxolitinib administration	July 1, 2020
**NCT04357730**	Fibrinolytic Therapy to Treat ARDS in the Setting of COVID-19 Infection	Recombinant tissue plasminogen activator with thrombolytic effect	• Drug: Alteplase 50 MG [Activase]	May 14, 2020
**NCT04350736**	First in Human SAD and MAD Study of Inhaled TD-0903, a Potential Treatment for ALI Associated With COVID-19	Nebulized JAK inhibitor	• Drug: TD-0903	April 23, 2020
**NCT04344184**	SAFEty Study of Early Infusion of Vitamin C for Treatment of Novel Coronavirus Acute Lung Injury (SAFE EVICT CORONA-ALI)	Anti-oxidant effect	• Drug: L-ascorbic acid	December 18, 2020
**NCT04340557**	Do Angiotensin Receptor Blockers Mitigate Progression to Acute Respiratory Distress Syndrome With SARS-CoV-2 Infection	Angiotensin II receptor blocker	• Drug: Losartan	March 27, 2020
**NCT04334629**	LIBERATE Trial in COVID-19	Non-steroidal anti-inflammatory drug	• Drug: Ibuprofen	May 26, 2020
**NCT04328467**	Pre-exposure Prophylaxis for SARS-Coronavirus-2	Treatment and prevention of malaria	• Drug: Hydroxychloroquine	April 6, 2020
**NCT04323592**	Methylprednisolone for Patients With COVID-19 Severe Acute Respiratory Syndrome	Corticosteroid	• Drug: Methylprednisolone	March 23, 2020
**NCT04321616**	The Efficacy of Different Anti-viral Drugs in COVID 19 Infected Patients	Treatment and prevention of malaria,	• Drug: Hydroxychloroquine• Drug: Remdesivir	March 28, 2020
**NCT04312009**	Losartan for Patients With COVID-19 Requiring Hospitalization	Angiotensin II receptor blocker	• Drug: Losartan	April 13, 2020
**NCT04311697**	Intravenous Aviptadil for Critical COVID-19 With Respiratory Failure	Synthetic vasoactive intestinal polypeptide	• Drug: Aviptadil by intravenous infusion + standard of care	May 15, 2020
**NCT04311177**	Losartan for Patients With COVID-19 Not Requiring Hospitalization	Angiotensin II receptor blocker	• Drug: Losartan	April 9, 2020
**NCT04308668**	Post-exposure Prophylaxis / Preemptive Therapy for SARS-Coronavirus-2	Treatment and prevention of malaria	• Drug: Hydroxychloroquine	March 17, 2020
**NCT04305457**	Nitric Oxide Gas Inhalation Therapy for Mild/Moderate COVID-19	Vasodilating agent by activation of guanylate cyclase	• Drug: Nitric Oxide	March 21, 2020
**NCT04954014**	Pilot Study of Single Dose Bevacizumab as Treatment for Acute Respiratory Distress Syndrome (ARDS) in COVID-19 Patients	Humanized monoclonal antibody against VEGF	• Drug: Bevacizumab	September 1, 2020
**NCT04745442**	Pilot Study of Antithrombin as Prophylaxis of Acute Respiratory Distress Syndrome in Patients With COVID-19	Antithrombin with anticoagulant effect	• Drug: Antithrombin	April 27, 2020
**NCT04640194**	A Study to Test Whether Different Doses of Alteplase Help People With	Recombinant tissue plasminogen activator with thrombolytic effect	• Drug: Alteplase	December 16, 2020

	Severe Breathing Problems Because of COVID-19			
**NCT04618042**	FX06 to Rescue Acute Respiratory Distress Syndrome During Covid-19 Pneumonia	Fibrin-derived peptide that stabilizes cell-cell interaction e.g. the endothelial barrier	• Drug: FX06	November 13, 2020
**NCT04615429**	Clinical Trial to Assess the Efficacy of MSC in Patients With ARDS Due to COVID-19	Mesenchymal stem cells	• Biological: Mesenchymal stromal cells	September 15, 2020
**NCT04609943**	Study to Find the Highest Safe Dose of Soluble Guanylate Cyclase (sGC) Activator, BAY 1211163 Administered as Multiple Doses by Inhalation to Patients Who Cannot Breathe by Their Own and Suffer From a Type of Lung Failure That Causes Fluid to Build up in the Lungs Making Breathing Difficult (ARDS)	Soluble guanylate cyclase activator	• Drug: BAY1211163 different doses	November 24, 2020
**NCT04586114**	Effect of Corticosteroid Treatment on Prognosis in ARDS Secondary to Covid-19	Corticosteroid	• Drug: Corticosteroids and Derivatives	March 1, 2020
**NCT04568018**	Surfactant-BL in Adult Acute Respiratory Distress Syndrome Due to COVID-19	Pulmonary surfactant	• Drug: Surfactant	July 3, 2020
**NCT04565249**	Evaluation of the Safety, Tolerability, and Pharmacokinetics of PLN-74809 in Participants With Acute Respiratory Distress Syndrome (ARDS) Associated With at Least Severe COVID-19	Small-molecule, dual selective inhibitor of αVβ1 / αVβ6 with anti-fibrotic action	• Drug: PLN-74809	October 22, 2020
**NCT04537806**	A Study of Brexanolone for Acute Respiratory Distress Syndrome Due to COVID-19	GABA A receptor-positive modulator	• Drug: Brexanolone	December 18, 2020
**NCT04537351**	The MEseNchymal coviD-19 Trial: MSCs in Adults With Respiratory Failure Due to COVID-19 or Another Underlying Cause	Mesenchymal stem cells	• Biological: CYP-001	August 24, 2020
**NCT04530604**	Defibrotide Therapy for SARS-CoV2 (COVID-19) Acute Respiratory Distress Syndrome (ARDS)	Oligonucleotide mixture with anti-thrombotic effects	• Drug: Defibrotide	October 1, 2020
**NCT04525378**	MSC-based Therapy in COVID-19-associated Acute Respiratory Distress Syndrome	Mesenchymal stem cells	• Other: Mesenchymal stromal cell-based therapy	July 1, 2020
**NCT04478071**	Vadadustat for the Prevention and Treatment of Acute Respiratory Distress Syndrome (ARDS) in Hospitalized Patients With Coronavirus Disease 2019 (COVID-19)	Hypoxia-inducible factor prolyl hydroxylase inhibitor, stimulated erythropoietin production	• Drug: vadadustat	August 22, 2020
**NCT04456361**	Use of Mesenchymal Stem Cells in Acute Respiratory Distress Syndrome Caused by COVID-19	Mesenchymal stem cells	• Biological: Mesenchymal Stem Cells derived from Wharton Jelly of Umbilical cords	April 16, 2020

**NCT04447833**	Mesenchymal Stromal Cell Therapy For The Treatment Of Acute Respiratory Distress Syndrome	Mesenchymal stem cells	• Drug: Mesenchymal Stromal Stem Cells- KI-MSC-PL-205	June 17, 2020
**NCT04442958**	Effectiveness of Convalescent Immune Plasma Therapy	Convalescent plasma	• Other: Convalescent Immune Plasma	May 15, 2020
**NCT04417036**	This Study Collects Information on the Safety of Inhaled Pegylated Adrenomedullin (PEG-ADM), How the Drug is Tolerated and How it Affects Patients Suffering From a Type of Lung Failure That Cause Fluid to Build up in the Lungs Making Breathing Difficult (ARDS)	Soluble guanylate cyclase activator with vasodilatory effect	• Drug: BAY1097761 two different doses	July 7, 2020
**NCT04416139**	Mesenchymal Stem Cell for Acute Respiratory Distress Syndrome Due for COVID-19	Mesenchymal stem cells	• Biological: Infusion IV of Mesenchymal Stem cells	May 1, 2020
**NCT04412057**	Clinical Trial to Evaluate CERC-002 in Adults With COVID-19 Pneumonia and Acute Lung Injury	Fully human monoclonal antibody against lymphotoxin-like, exhibits inducible expression, and competes with glycoprotein D for HVEM, a receptor expressed by T cells	• Drug: CERC-002	June 9, 2020
**NCT04408326**	Efficacy and Safety of Angiotensin II Use in Coronavirus Disease(COVID)-19 Patients With Acute Respiratory Distress Syndrome	Angiotensin II with vasoconstrictory and pro-inflammatory action; anti-inflammatory action	• Drug: Angiotensin II• Drug: Interleukin-1 receptor antagonist	June 1, 2020
**NCT04400032**	Cellular Immuno-Therapy for COVID-19 Acute Respiratory Distress Syndrome	Mesenchymal stem cells	• Biological: Mesenchymal Stromal Cells	May 15, 2020
**NCT04390152**	Safety and Efficacy of Intravenous Wharton's Jelly Derived Mesenchymal Stem Cells in Acute Respiratory Distress Syndrome Due to COVID 19	Mesenchymal stem cells	• Drug: Wharton's jelly derived Mesenchymal stem cells• Drug: Hydroxychloroquine, lopinavir/ ritonavir or azithromycin and placebo (standard therapy)	January 13, 2020
**NCT04389671**	The Safety and Preliminary Tolerability of Lyophilized Lucinactant in Adults With Coronavirus Disease 2019 (COVID-19)	Pulmonary surfactant	• Drug: Lucinactant	November 2, 2020
**NCT04384731**	Curosurf® in Adult Acute Respiratory Distress Syndrome Due to COVID-19	Pulmonary surfactant	• Drug: poractant alfa	May 29, 2020
**NCT04378920**	A Study of Liposomal Trans Crocetin, LEAF-4L6715, in Patients With Acute Respiratory Distress Syndrome Due to COVID-19, Sepsis or Other Causes	NMDA receptor antagonist	• Drug: LEAF-4L6715	April 14, 2020
**NCT04371393**	MSCs in COVID-19 ARDS	Mesenchymal stem cells	• Biological: Remestemcel-L	April 30, 2020

**NCT04369469**	Efficacy and Safety Study of IV Ravulizumab in Patients With COVID-19 Severe Pneumonia	Humanized monoclonal antibody against complement C5	• Biological: Ravulizumab	May 11, 2020
**NCT04366063**	Mesenchymal Stem Cell Therapy for SARS-CoV-2-related Acute Respiratory Distress Syndrome	Mesenchymal stem cells	• Biological: Cell therapy two protocols	April 5, 2020
**NCT04357444**	Low Dose of IL-2 In Acute Respiratory DistrEss Syndrome Related to COVID-19	IL-2 for immune homeostasis, anti-tumor agent	• Drug: 1: ILT101	October 23, 2020
**NCT04355364**	Efficacy and Safety of aerosolizedDornase Alfa Administration in Patients With COVID19 Induced ARDS (COVIDORNASE)	Dornase Alfa	• Drug: Dornase Alfa Inhalation Solution [Pulmozyme]	April 21, 2020
**NCT04351243**	A Study to Assess the Efficacy and Safety of Gimsilumab in Subjects With Lung Injury or Acute Respiratory Distress Syndrome Secondary to COVID-19 (BREATHE)	Monoclonal antibody against granulocyte macrophage colony stimulating factor	• Drug: Gimsilumab	April 12, 2020
**NCT04350580**	Polyvalent Immunoglobulin in COVID-19 Related ARds	Immunoglobulin	• Drug: Human immunoglobulin	April 11, 2020
**NCT04347980**	Dexamethasone Treatment for Severe Acute Respiratory Distress Syndrome Induced by COVID-19	Corticosteroid, anti-malaria drug	• Drug: Dexamethasone and Hydroxychloroquine	April 2020
**NCT04335786**	Valsartan for Prevention of Acute Respiratory Distress Syndrome in Hospitalized Patients With SARS-COV-2 (COVID-19) Infection Disease	Angiotensin II receptor blocker	• Drug: Valsartan (Diovan)	April 17, 2020
**NCT04333368**	Cell Therapy Using Umbilical Cord-derived Mesenchymal Stromal Cells in SARS-CoV-2-related ARDS	Mesenchymal stem cells	• Biological: Umbilical cord Wharton's jelly-derived human	April 6, 2020
**NCT04331613**	Safety and Efficacy of CAStem for Severe COVID-19 Associated With/Without ARDS	Mesenchymal stem cells	• Biological: CAStem	January 27, 2020
**NCT03969992**	A Dose-Ranging Study to Determine the Efficacy, Safety and Tolerability of AeroFact	Pulmonary surfactant	• Drug: AeroFact• Other: nCPAP	March 4, 2020
**NCT04606563**	Host Response Mediators in Coronavirus (COVID-19) Infection - Is There a Protective Effect of Losartan on Outcomes of Coronavirus Infection?	Angiotensin II receptor blocker	• Drug: Losartan	October 9, 2020
**NCT04603924**	Study of Niclosamide in Moderate and Severe Hospitalized Coronavirus-19 (COVID-19) Patients	Multi-functional antihelminthic drug	• Drug: Niclosamide	October 7, 2020
**NCT04576728**	Efficacy and Safety of Trimodulin in Subjects With Severe COVID-19	Polyvalent antibody composition, purified from human plasma, containing IgG, IgM and IgA	• Drug: Trimodulin	October 6, 2020
**NCT04494724**	Clazakizumab vs. Placebo - COVID-19 Infection	Glycosylated humoral rabbit monoclonal antibody against IL-6	• Drug: Clazakizumab	July 13, 2020

**NCT04482699**	RAPA-501-Allo Therapy of COVID-19-ARDS	Allogeneic Hybrid TREG/Th2 Cell T-cells extracted from healthy volunteers with anti-inflammatory action	• Biological: RAPA-501-Allo off-the-shelf Therapy of COVID-19	December 30, 2020
**NCT04459325**	Tigerase® Efficacy and Safety as Part of Complex Therapy in Patients With COVID-19	Dornase alpha	• Biological: Tigerase® and best available care	June 1, 2020
**NCT04412668**	Study to Evaluate the Safety and Efficacy of ATYR1923 In Patients With Severe Pneumonia Related to COVID-19	Selective NRP2 modulator that downregulates innate and adaptive immune responses	• Drug: ATYR1923 1 and 3 mg/kg	June 12, 2020
**NCT04382755**	Zilucoplan® in Improving Oxygenation and Short- and Long-term Outcome of COVID-19 Patients With Acute Hypoxic Respiratory Failure	Complement 5 inhibitor	• Drug: Zilucoplan®	May 22, 2020
**NCT04357782**	Administration of Intravenous Vitamin C in Novel Coronavirus Infection (COVID-19) and Decreased Oxygenation	Anti-oxidant effect	• Drug: L-ascorbic acid	April 16, 2020
**NCT04330638**	Treatment of COVID-19 Patients With Anti-interleukin Drugs	Recombinant IL-1 receptor antagonist; different humanized monoclonal antibody against IL-6	• Drug: Anakinra• Drug: Siltuximab• Drug: Tocilizumab	April 3, 2020
**NCT04326920**	Sargramostim in Patients With Acute Hypoxic Respiratory Failure Due to COVID-19 (SARPAC)	Recombinant human granulocyte-macrophage colony stimulating factor that increases immune cell production	• Drug: Sargramostim	March 24, 2020
**NCT04315298**	Evaluation of the Efficacy and Safety of Sarilumab in Hospitalized Patients With COVID-19	Monoclonal IL-6 receptor antibody	• Drug: Sarilumab	March 18, 2020
